# Electrostatic Doping of 2D Semiconductors Using Charged Dielectric Thin Films

**DOI:** 10.1021/acsnano.6c07356

**Published:** 2026-07-15

**Authors:** Xinya Niu, John O’Sullivan, Bin Han, Lixin Liu, Yi Cui, Yan Wang, Manish Chhowalla, Ruy S. Bonilla

**Affiliations:** † Department of Materials, 6396 University of Oxford, Oxford OX1 3PH, U.K.; ‡ Oxford Suzhou Centre for Advanced Research (OSCAR), University of Oxford, Suzhou 215123, China; § 85404 Suzhou Institute of Nano-tech and Nano-bionics, Chinese Academy of Sciences, Suzhou 215123, China; ∥ 27083 University of Strasbourg, CNRS, ISIS, UMR 7006, Strasbourg F-67000, France; ⊥ Department of Materials Science & Metallurgy, 2152 University of Cambridge, Cambridge CB3 0FS, U.K.

**Keywords:** two-dimensional semiconductors, electrostatic doping, dielectric defect engineering, interface states, MoS_2_

## Abstract

Doping two-dimensional (2D) semiconductors without direct chemical or structural modification of the channel remains a central challenge for device integration. Here we demonstrate an electrostatic doping strategy on monolayer MoS_2_ based on embedding fixed charge in engineered dielectric stacks, enabling carrier modulation in the absence of volatile external bias. By comparing different dielectric architectures, we show that effective electrostatic doping is governed by the defect landscape of the capping dielectrics and their interface with the 2D channel. A self-consistent electrostatic model reveals that interface states control the partitioning of the dielectric embedded charge between carriers trapped in defects or free for conduction in the channel, posing limits to the effectiveness of electrostatic coupling. This work establishes electrostatic doping as a viable strategy for carrier modulation in 2D semiconductors and identifies dielectric defect engineering as central to its implementation.

## Graphical abstract

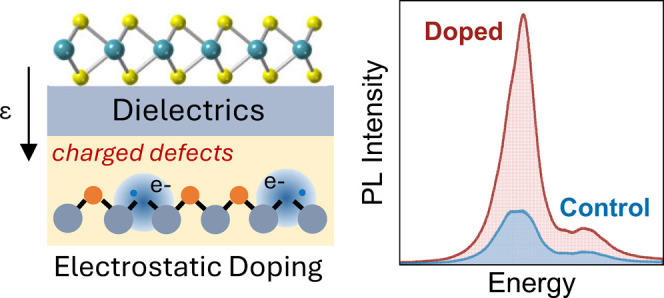

Doping is essential for enabling functional devices based on two-dimensional (2D) materials, as it allows precise control over electronic properties such as carrier type and concentration. Conventional strategies, including ion implantation,^1^ bottom-up substitutional doping,^2–4^ and charge transfer from molecular adsorbates,^5–8^ have been widely explored but face key limitations. These approaches often lack long-term stability,^5^ offer limited control over spatial selectivity,^9^ and tend to degrade carrier mobility due to increased scattering.^5,10^ In comparison, electrostatic gating fully preserves the channel structure and thus the carrier mobility. While highly effective, traditional gating requires electrodes and continuous external bias, which makes it unsuitable for energy-efficient and versatile applications in electronic devices.

To address the challenges of conventional doping techniques, electrostatic doping has emerged as a contactless approach to modulate carrier density in 2D materials by electrostatically coupling fixed charges in nearby dielectrics. Previous work has demonstrated reversible carrier-density modulation through corona-charge deposition on membranes integrated with graphene prepared by chemical vapor deposition (CVD).^11^ Another demonstrated strategy involves the use of solid state ions, such as K^+^ in thermally grown SiO_2_, which migrate under an applied electric field and induce reversible doping in adjacent CVD-grown 2D MoS_2_ layers.^12^ This method enables field-induced charge modulation without introducing structural defects, but requires elevated temperatures to activate ion motion, limiting its applicability in low energy consumption devices. Alternative approaches based on charge transfer from dielectric defects have been explored, where external stimuli such as electron beam irradiation or intense light pulses generate trapped charges within dielectric layers.^13–15^ These trapped charges can modulate carrier densities in the underlying 2D material and have been used to achieve high doping levels and patterned doping.^13^ However, the charging process generally relies on vacuum-based setups and high-energy sources, making it less suitable for scalable or ambient-compatible integration.

In this work, we present an electrostatic doping strategy for monolayer MoS_2_, that preserves the structural integrity and intrinsic mobility of the 2D channel. Stable negative charges are embedded several nanometres beneath the surface of a dielectric thin film, through a simple, ambient-air hot-corona charging process, eliminating the need for high-energy or vacuum-based treatments. These embedded charges generate a built-in electric field that modulates the carrier density in the neighboring MoS_2_ layer, achieving nondestructive and air-stable doping.

The concept was first validated using corona-charged poly­(methyl methacrylate) (PMMA) films, which enable reversible modulation of carrier density in MoS_2_ but suffer from poor charge retention. A newly developed charged SiO_2_/HfO_*x*_ thin film stack provides long-term charge stability, yielding a reduction in electron density, as demonstrated by both transport measurements and PL spectroscopy. By comparing multiple charged dielectric systems, we identify the defect landscape in the capping dielectric and its interface to the 2D channel as the key electrical parameters governing the effectiveness electrostatic doping. This work offers a controllable route toward nonvolatile, mobility-preserving doping of 2D semiconductors.

## Results

### Doping from a Charged PMMA Top Dielectric

To demonstrate the feasibility of electrostatic doping in 2D semiconductors using charged dielectrics, a PMMA layer of ∼500 nm was deposited on a back-gate field-effect transistor (FET) with a monolayer MoS_2_ channel. The schematic of the device structure is shown in Figure 1a. Corona charges were deposited at room temperature under ambient conditions in 20 s intervals. The first 80 s corresponds to successive negative corona charge deposition, followed by 60 s of successive positive corona charging. Following each deposition, the net surface charge density (*Q*_surf_/*q*, where q is the elementary charge) was monitored using a Kelvin probe,^16^ while the induced carrier density (*n*_el_) in the 2D MoS_2_ channel was quantified from transfer characteristics. Figure 1b,c display the evolution of the transfer curves with cumulative charging. The extracted *n*_el_ and *Q*_surf_/*q* values at each step are presented in Table S1. The absolute current levels are relatively low, which is consistent with contact-limited transport arising from nonoptimized Al contacts.^17,18^

**Figure 1:**
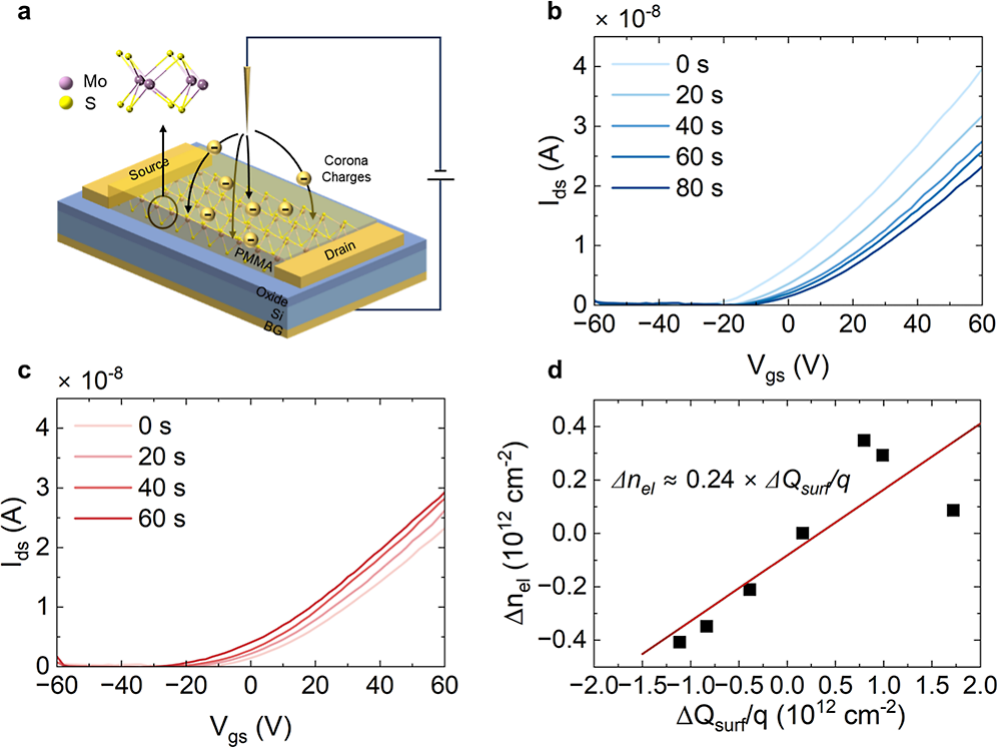
(a) Schematic illustration of the back-gated field-effect transistor (FET) using a monolayer MoS_2_ channel, where a PMMA layer serves as a top dielectric to retain corona charges. (b,c) Transfer characteristics of the device under progressively increasing (b) negative and (c) positive corona charge deposition with *V*_ds_ = 0.01 V. (d) Change in channel electron density (Δ*n*_el_) as a function of the deposited surface charge density (Δ*Q*_surf_/*q*).

A clear increase in the threshold voltage (*V*_th_) was observed with the deposition of negative corona charge, followed by a recovery after subsequent positive corona charging. This behavior demonstrates effective modulation of the channel carrier density through control of both the polarity and magnitude of the surface charge density. To quantify this relationship, the change in electron density in the MoS_2_ channel following each corona charge deposition (Δ*n*_el_) was analyzed as a function of the deposited surface charge density (Δ*Q*_surf_/*q*) and shown in Figure 1d. The linear fit indicates that approximately 24% of the deposited surface charge contributes to modulating the channel carrier density. The deviation from ideal one-to-one coupling can be partially attributed to the limited stability of corona charges on the PMMA layer, as demonstrated in Figure S1. In addition, defect states at the MoS_2_/PMMA interface are expected to screen the electric field. An interface defect density of approximately 9 × 10^11^ cm^–2^ eV^–1^ has been reported for PMMA-encapsulated MoS_2_,^19^ providing defect states that can respond to the applied electric field and reduce its effective coupling to the carrier population in the channel.

### Design and Fabrication of Negatively Charged Oxide Nanolayers

The previous section demonstrated a proof of concept for manipulating the carrier density of a 2D channel by charged dielectrics using PMMA with deposited corona charges. However, the charges stored in PMMA suffer from poor long-term stability due to lack of deep states,^20^ which requires an alternative approach with enhanced charge stability.

To address this limitation, we propose embedding deep-level defects into ALD-deposited, high quality oxide thin films to enable stable negative charge storage while maintaining full compatibility with 2D material devices.^21,22^ In such oxides, deep states act as effective charge traps, suppressing charge relaxation and thereby improving stability.^23,24^ Based on this mechanism, two negatively charged dielectric stacks were designed and evaluated. Schematic diagrams of both dielectric stacks and the corresponding charging strategies are shown in Figure 2a,d, respectively. Stack A employs an engineered defect layer followed by a separate charging step, whereas Stack B utilizes a charging strategy that simultaneously creates and charges the defects. Capping layers are introduced to physically separate the charged defects from the 2D channel to prevent direct interactions. This design ensures that the influence of the charged dielectric on the 2D channel is predominantly electrostatic, rather than arising from charge transfer, while minimizing Coulomb scattering.^25–27^

**Figure 2:**
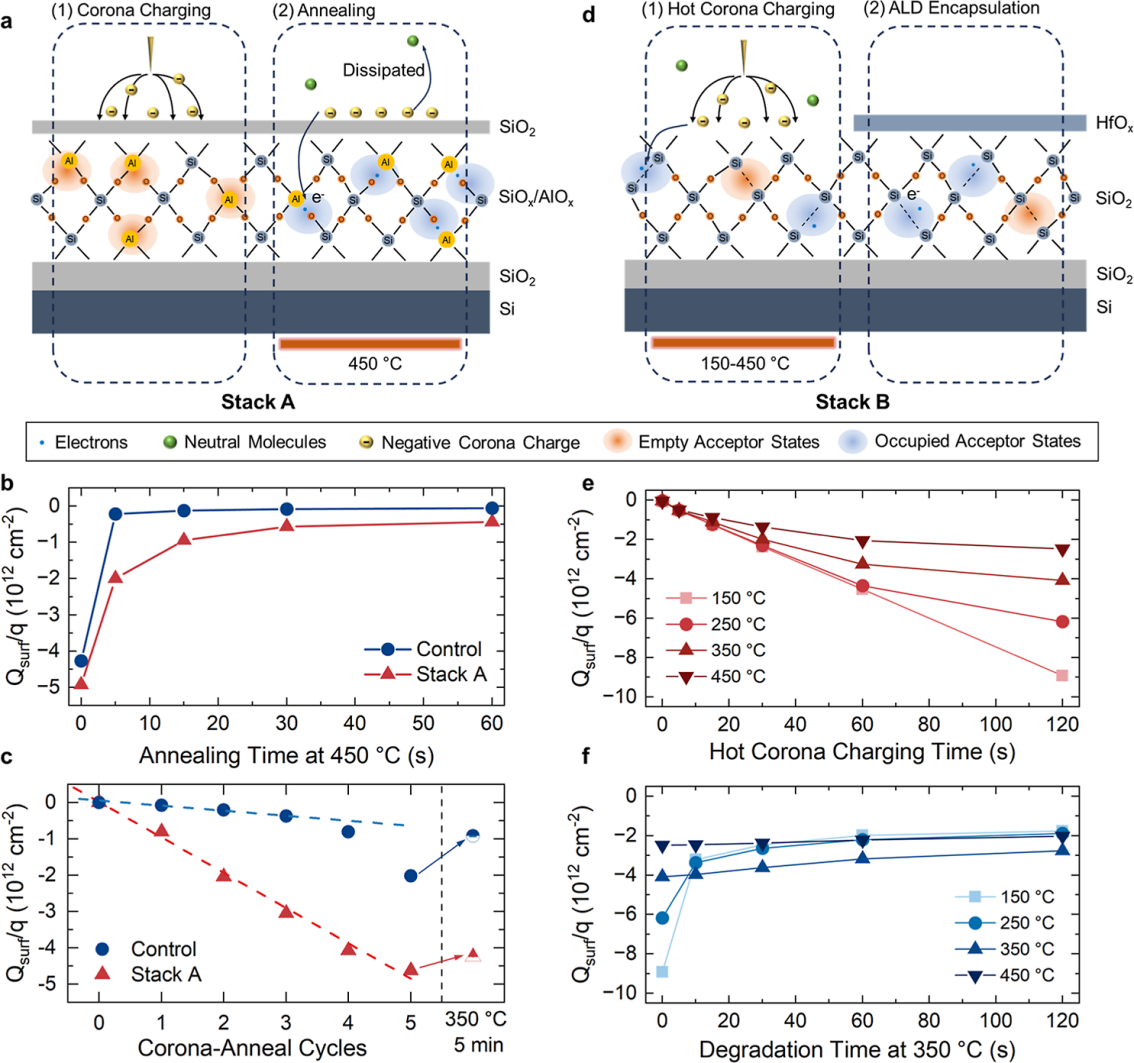
(a) Schematic illustration of the charging mechanism of Stack A using corona-annealing. Deep acceptor states are formed at the SiO_*x*_/AlO_*x*_ interface. Following the deposition of negative air ions at room temperature, an annealing at 450 °C facilitates electron injection into the deep acceptor states while part of the negative air ions dissipates. (b) Evolution of surface charge density *Q*_surf_/*q* as a function of annealing time after corona charge deposition, comparing to a control SiO_2_ stack and Stack A. (c) Accumulation of negative *Q*_surf_/*q* with repeated corona-anneal cycles. The rightmost data points correspond to the same samples after an additional anneal at 350 °C for 5 min to demonstrate charge thermal stability. (d) Schematic illustration of charging mechanism of Stack B using hot corona charging and subsequent ALD encapsulation. (e) *Q*_surf_/*q* as a function of hot corona charging time at different substrate temperatures. (f) Thermal degradation of stored negative charge at 350 °C following hot corona charging at different temperatures, indicating increased charge stability for higher charging temperatures.

Stack A exploits deep acceptor states at the SiO_*x*_/AlO_*x*_ interface, which have been extensively reported in the context of silicon solar cell passivation,^28–33^ but have not yet been employed for electrostatically modulating charge carriers in 2D material devices. These interface defect states are charged externally by depositing negative corona charge at room temperature, during which ionised air species (e.g., CO_3_^–^) are proposed to transfer excess electrons to the defect states.^34^

A targeted 25 nm (360 ALD cycles) thick ALD-SiO_*x*_ layer was used as the capping layer, which allows both efficient charge injection at 450 °C and maintains charge stability at low temperatures. The optimization of the SiO_*x*_ capping layer thickness is shown in Figure S2. To inject electrons into the buried SiO_*x*_/AlO_*x*_ interface states, a corona-anneal method was employed as described in the Methods section. Elevated temperatures both facilitate charge injection into deep defect states by providing sufficient thermal energy to overcome energy barriers, and accelerate charge detrapping process, making high-temperature measurements a stringent test of charge stability. Reduced charge stability at elevated temperatures is demonstrated in Figure S3.

To evaluate the thermal stability of the injected charges, a negative surface charge density of approximately 4–5 × 10^12^ q cm^–2^ was first deposited on the sample surface, followed by annealing at 450 °C. The evolution of the effective surface charge density was monitored using Kelvin probe measurements and the calculated *Q*_surf_/*q* is shown in Figure 2b. For comparison, a control sample without the AlO_*x*_ layer was also tested, which exhibited rapid charge loss within 5 s of annealing. Meanwhile, Stack A demonstrated a stable negative charge concentration of ∼7.2 × 10^11^ q cm^–2^ after 60 s of annealing. The pronounced contrast indicates that deep acceptor states at the SiO_*x*_/AlO_*x*_ interface can provide effective trapping sites that stabilize negative charges under thermal stress.

To assess the reproducibility and maximum achievable charge density of this approach, repeated corona-anneal cycles were performed on Stack A and the control sample. The evolution of *Q*_surf_/*q* with increasing charging cycles is shown in Figure 2c. A linear increase in the magnitude of *Q*_surf_/*q* was observed with successive charging cycles, yielding an average charging rate of 8.6 × 10^11^ q cm^–2^ per cycle for Stack A. This is significantly higher than that measured for the control sample without the AlO_*x*_ layer. Following charge injection, the samples are annealed at 350 °C for 5 min, with minimal degradation in negative *Q*_surf_/*q* in Stack A.

In contrast, the small but finite slope observed in the control samples suggests the gradual formation of chargeable defects during repeated charging cycles, which are likely associated with near-surface states in the ALD-SiO_*x*_.^35,36^ To minimize the creation of uncontrolled surface defects that could affect device integration, the number of corona-anneal cycles was limited to four for samples used in subsequent 2D device demonstrations.

Stack B relies on defect states created and charged during high-temperature corona charging, which has been reported to yield highly stable charged dielectrics by facilitating charge penetration deeper into the dielectric bulk.^37,38^ To evaluate the maximum achievable charge density and its thermal stability, corona charging was performed while heating the Si substrate with 300 nm thermal SiO_2_ to temperatures between 150 and 450 °C, followed by stability tests at 350 °C. The evolution of *Q*_surf_/*q* during charging process is shown in Figure 2e, while changes in *Q*_surf_/*q* during degradation are shown in Figure 2f. During charging, lower substrate temperatures (150 and 250 °C) resulted in higher negative *Q*_surf_/*q* but degraded rapidly during subsequent thermal stress at 350 °C, ultimately falling below the *Q*_surf_/*q* levels obtained from samples charged at higher temperatures. This result indicates that charge injected at elevated temperatures is more thermally robust, consistent with populating of defect states deeper within the dielectric bulk.

We propose that one of the key requirements for designing charge-retaining dielectrics is the presence of defect states with deep energy levels. The two defect configurations investigated in this work are (i) acceptor-like states at the SiO_*x*_/AlO_*x*_ interface, and (ii) defects generated near the SiO_2_ surface during hot corona charging. Such defects may originate intrinsically from dielectric intermixing at interfaces,^30,39–41^ disorder within amorphous oxide networks,^42,43^ or externally induced bond breaking.^36,44^

For the SiO_*x*_/AlO_*x*_ interface, previous studies have suggested that oxygen-vacancy-related defects, oxygen dangling bonds, and negatively charged AlO_4_ configurations are likely contributors to deep acceptor-like trap states near the valence band of AlO_*x*_.^41,42,45^ Defects generated during hot corona charging could be associated with oxygen-related dangling bonds and weak or broken Si–O bonds formed under energetic charging conditions, introducing deep localized trapping states within the SiO_2_ bandgap.^46,47^

A capping layer deposited after charging is proposed here to prevent direct interactions between the charged defect layer and the 2D channel. Silicon substrates with 300 nm thermal SiO_2_, charged at 450 °C for varying durations, were capped with either ALD-grown SiO_*x*_ or HfO_*x*_. As shown in Table S2, samples capped with HfO_*x*_ retained a substantially larger fraction of the injected negative charge than those capped with SiO_*x*_. This observation is attributed to the lower effective defect density of HfO_*x*_, which limits charge dissipation pathways.^48^ Some charge loss was observed for both capping layers, likely due to exposure to ALD precursor gases during the initial stages of film growth. Further evidence of the strong charge-retention capability of a 5 nm layer of HfO_*x*_ is presented in Figure S4.

### Electrostatic Doping of MoS_2_ via Charged Dielectric Nanolayers

To evaluate the modulation of carrier densities in 2D semiconductors using charged dielectrics, monolayer MoS_2_ field-effect transistors (FETs) were fabricated on Stack A and Stack B and compared to uncharged control substrates with identical dielectric structures. Carrier modulation was assessed using electrical transport measurements on completed devices, complemented by PL spectroscopy on transferred 2D MoS_2_ flakes without device fabrication. Schematics illustrations of 2D MoS_2_ on Stack A and B and their corresponding control groups are shown in Figure 3a,d, respectively. The transfer curves and the extracted threshold voltage (*V*_th_) devices on both charged stacks and their control groups are shown in Figure 3b,e, while the PL spectra and the extracted trion-to-exciton integrated intensity ratios (in *I*_A_–__/*I*_A0_) across multiple flakes are shown in Figure 3c,f. Transfer curves are normalized to enable comparison across devices with different channel dimensions. Optical images, complete transfer characteristics, and details of parameter extraction, as well as device-to-device variations in *Q*_surf_/*q*, are provided in Supporting Information.

**Figure 3:**
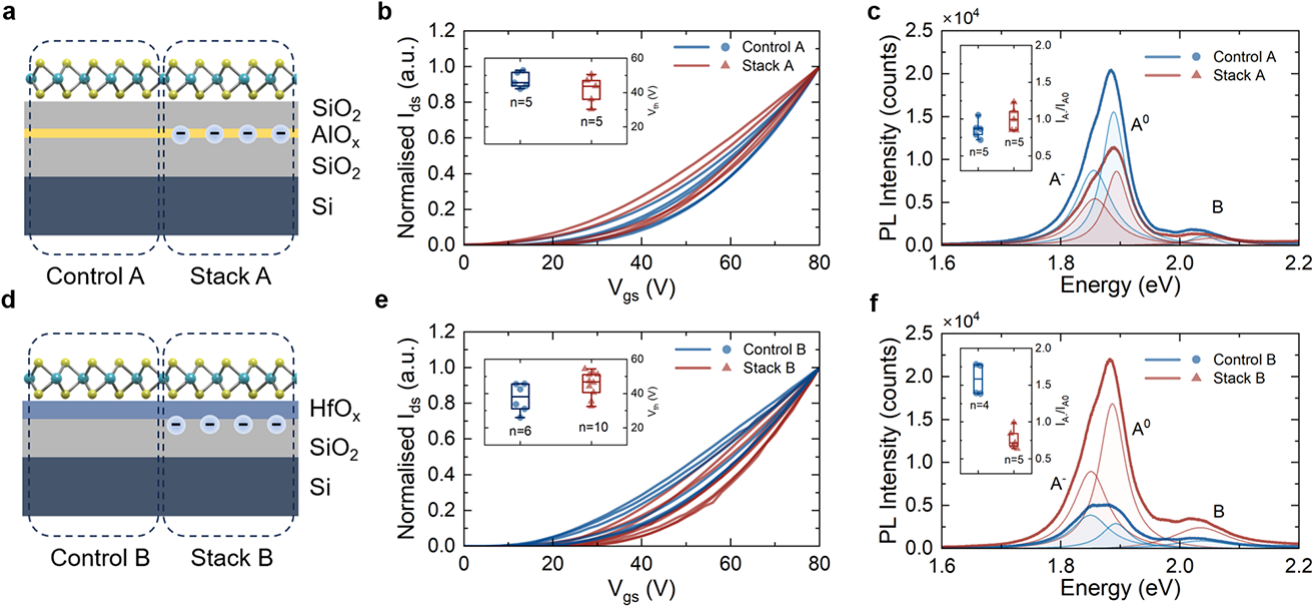
(a,d) Schematic cross sections of MoS_2_ on Stack A (SiO_*x*_ 28 nm/AlO_*x*_ 2 nm/SiO_2_ 300 nm/Si) and B (HfO_*x*_ 5 nm/SiO_2_ 300 nm/Si) and their corresponding control groups. Negative charges are embedded at the SiO_2_/AlO_*x*_ interface and near the SiO_2_ surface for Stack A and B, respectively. (b,e) Normalized transfer characteristics (normalized to *I*_ds_ at *V*_gs_ = 80 V) of MoS_2_ FETs fabricated on (b) Control A and Stack A and (e) Control B and Stack B. Each line corresponds to an individual device. Insets show extracted *V*_th_ obtained by linear extrapolation of the transfer curves over *V*_gs_ = 70–80 V range with sample sizes indicated. (c,f) PL spectra of monolayer MoS_2_ flakes with representative in *I*_A_–__/*I*_A0_ values for (c) Control A and Stack A and (f) Control B and Stack B. Insets show extracted trion-to-exciton integrated intensity ratios (in *I*_A_–__/*I*_A0_) obtained from Lorentz peaking fitting with sample sizes indicated.

We first examine carrier modulation in MoS_2_ devices fabricated on Stack A. Stack A was charged using a corona-anneal process prior to MoS_2_ transfer, resulting in a *Q*_surf_/*q* of −(3.6–4.4) × 10^12^ cm^–2^, as determined by Kelvin probe measurements. As shown in Figure 3b, despite the substantial fixed charge embedded in the substrate, MoS_2_ devices on Stack A exhibit no statistically significant shift in *V*_th_ relative to uncharged controls. Instead, an increase in hysteresis (Figure S21) and enhanced device-to-device variability was observed, consistent with increased interfacial charge trapping rather than effective electrostatic doping of the 2D MoS_2_ channel.^49–51^ Such additional defects may be introduced in the SiO_2_ capping layer during the corona-anneal process, as suggested by the increased charging rate observed for control samples in Figure 2c. Meanwhile, *μ*_FE_ remains comparable between charged and uncharged substrates, indicating preserved channel quality. As shown in Figure 3c, MoS_2_ on Stack A exhibits PL features similar to uncharged controls, with no statistically significant difference in in *I*_A_–__/*I*_A0_. However, the two independent measurements consistently indicate a weak trend toward n-type doping behavior on Stack A compared to Control A.

In contrast to Stack A, MoS_2_ devices fabricated on Stack B exhibit clear carrier modulation compared to controls, as shown in Figure 3e. Stack B was charged prior to HfO_*x*_ deposition and device fabrication, resulting in a *Q*_surf_/*q* of −(5.1–6.5) × 10^12^ cm^–2^ as determined by Kelvin probe measurements.

Photoluminescence spectroscopy reveals statistically significant carrier modulation on Stack B compared to Control B. As shown in Figure 3f, MoS_2_ flakes on Stack B exhibit a pronounced enhancement of the neutral A exciton (A^0^) peak and a corresponding suppression of the negatively charged trion (A^–^) peak, resulting in a reduction in *I*_A_–__/*I*_A0_, as shown in the inset of Figure 3f. Transport measurements demonstrated a positive shift in *V*_th_ on devices on Stack B compared to Control B. Although this shift does not reach statistical significance, it is consistently in the same direction as the PL response and indicates a reduction in electron density in the MoS_2_ channel. The magnitude of the average *V*_th_ shift corresponds to an effective reduction in *n*_el_ of approximately 4.8 × 10^11^ cm^–2^. Taken together, the transport and PL results establish effective electrostatic doping of monolayer MoS_2_ on Stack B.

Importantly, as HfO_*x*_ was deposited after charging, the charged and uncharged samples are expected to have similar HfO_*x*_ surface defect conditions and comparable defect-channel charge transfer. Any additional defects introduced by the charging process are confined to the underlying SiO_2_ and spatially separated from the MoS_2_ channel by the HfO_*x*_ layer where charge transfer is suppressed,^52^ indicating that the observed reduction in *n*_el_ is predominantly an electrostatic effect rather than direct charge transfer. Consistent with this interpretation, the field-effect mobility (*μ*_FE_) remains comparable between Stack B and Control B (Figure S21), indicating that carrier modulation is achieved without measurable degradation of channel transport.

To further test the role of the charging sequence, hot corona charging was applied to a Control B substrate, in which the HfO_*x*_ capping layer was deposited prior to charging. Monolayer MoS_2_ flakes transferred onto these substrates show no measurable change in photoluminescence (Figure S22), indicating the absence of carrier modulation. This observation highlights the critical role of the charging sequence, suggesting that effective electrostatic coupling is achieved only when charge embedding precedes deposition of the HfO_*x*_ capping layer.

## Discussion

Electrostatic doping of 2D MoS_2_ is observed only for specific charged dielectric stacks, despite comparable *Q*_surf_/*q*. Carrier modulation is observed on charged PMMA and on substrate in which charge embedding precedes capping layer deposition (Stack B), whereas Stack A and substrate charged after capping layer deposition (hot corona charged Control B) fail to induce measurable doping. These results indicate that the presence of fixed charge in the dielectric alone does not guarantee electrostatic doping of 2D channel.

These observations can be rationalized by considering the stability of embedded charges and the existence of energetically accessible charge-relaxation pathways. When such a pathway is present, injected charges may relax through defect-mediated processes, thereby reducing the built-in electric field at the 2D channel. This relaxation process is expected to become possible once MoS_2_ is introduced and electronic equilibration can occur between the charged defects and the semiconductor, when defects states lie above the MoS_2_ Fermi level. Such relaxation may be facilitated by thermal processing during device fabrication such as thermal evaporation, or during transport measurements. Under these conditions, electrostatic modulation of the MoS_2_ channel remains limited despite a large embedded dielectric charge density.

This interpretation provides a plausible explanation for the lack of electrostatic doping observed in Stack A. Although the SiO_2_ capping layer thickness was optimized to suppress charge degradation at room temperature (Figure S2), this optimization was performed on low-defect SiO_2_. The corona-anneal process applied is likely to generate additional defects, as suggested by the increased charging rate observed in Figure 2c and the increased hysteresis (*V*_hys_) observed on Stack A devices, as shown in Figure S21. These defects are most likely to be oxygen-vacancy-related states and are known to have a broad distribution of energy levels inside of the SiO_2_ bandgap.^36^ Such states can form energetically accessible pathways that facilitate charge relaxation from buried acceptor states at the SiO_*x*_/AlO_*x*_ interface. A schematic band diagram illustrating this process is shown in Figure 4a.

**Figure 4:**
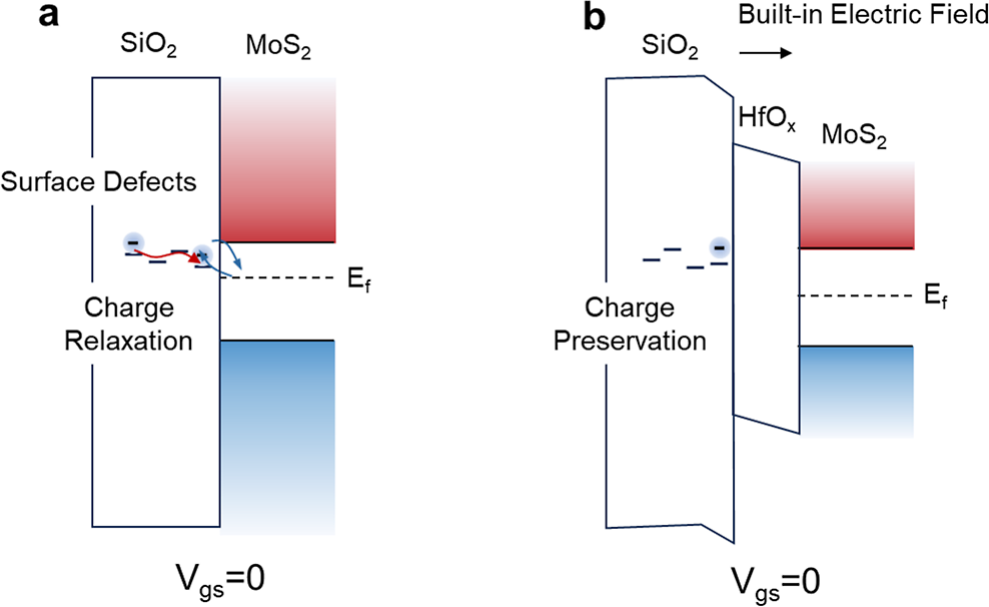
Schematic band diagrams under zero back gate voltage showing (a) charge relaxation at room temperature through surface defect states in the SiO_2_ capping layer and n-type doping of 2D MoS_2_ induced by surface defects. (b) Charge preservation and effective electrostatic doping enabled by a low-defect HfO_*x*_ capping layer.

The n-type trend observed in 2D MoS_2_ on Stack A compared with the Control A substrate is consistent with enhanced interactions between the 2D channel and dielectric surface defects. Prior studies have shown that charged defect states at the SiO_2_ can shift the Fermi level of MoS_2_, leading to n-type behavior.^27,53^ N-type doping has also been reported for MoS_2_ on plasma-treated SiO_2_ surfaces, where the formation of chemically active oxygen bonds and its interfacial bonding has been proposed to promote electron accumulation in the MoS_2_ layer.^54^

In contrast, robust electrostatic doping is achieved when such charge-relaxation pathways are mitigated. In the case of charged PMMA, corona charging is performed after PMMA deposition, although surface defects may be generated during charge deposition,^36^ the large PMMA thickness (∼500 nm) strongly limits defect penetration toward the MoS_2_ channel, allowing effective carrier modulation. Similarly, in Stack B, charge embedding precedes deposition of the HfO_*x*_ capping layer, resulting in a low-defect dielectric that effectively suppresses charge relaxation (Figure S4) and preserves electrostatic coupling. In contrast, when the charging sequence is reversed and hot corona charging is applied after HfO_*x*_ deposition, defects are introduced directly into the HfO_*x*_ layer (Figure S5), forming energetically accessible charge-relaxation pathways.

Thermionic emission and direct tunnelling are unlikely to dominate the observed charge relaxation. Despite exhibiting poorer charge retention, SiO_*x*_ has a larger bandgap (9 eV) than HfO_*x*_ (5.8 eV), which provides a higher thermionic emission barrier. In addition, both capping layers are thicker than 5 nm, whereas direct tunnelling becomes significant mainly in ultrathin dielectrics below ∼2 nm due to the exponential distance dependence of tunnelling probability.^55^ Calculated thermionic and tunnelling probability is included in Supporting Information. These observations instead suggest that defect-mediated charge relaxation is the dominant mechanism. A schematic band diagram of this process is shown in Figure 4b.

To compare the charge-retention characteristics of the commonly used dielectrics SiO_*x*_, AlO_*x*_, and HfO_*x*_, corona-annealing experiments were performed, with results shown in Figure S5. The measurements indicate that HfO_*x*_ most effectively preserves the embedded charge within the buried SiO_*x*_/AlO_*x*_ layer, followed by SiO_*x*_ and AlO_*x*_. This result highlights the critical role of dielectric-dependent charge-relaxation pathways in determining long-term electrostatic doping stability.

For Stack B, transport and PL measurements yield substantially different estimates of the change in carrier density compared to Control B (Δ*n*_el_). Transport measurements indicate a modest Δ*n*_el_ of ∼−4.8 × 10^11^ q cm^–2^, extracted from the average *V*_th_ shift, whereas PL analysis suggests a much larger apparent Δ*n*_el_ of −2.62 × 10^13^ q cm^–2^, inferred from change in the average in *I*_A_–__/*I*_A0_. Both values differ from the embedded *Q*_surf_/*q* of −(5.1–6.5) × 10^12^ cm^–2^.

This discrepancy reflects the distinct carrier populations and physical processes probed by transport and PL measurements. Transport measurements are sensitive only to mobile carriers that contribute to electrical conduction, which can be suppressed by interface trap states or nonideal metal-channel contacts,^56–58^ resulting in only a moderate change in the extracted *n*_el_ in this work. In contrast, PL is sensitive to local shifts of the Fermi level and to changes in nonradiative recombination pathways.^59–61^ In Stack B, the embedded charges reduce the electron population near the interface and passivate interface trap states and suppress nonradiative recombination, leading to a larger apparent *n*_el_ estimated from the variation of in *I*_A_–__/*I*_A0_. To obtain a more reliable estimate of the charge modulation induced by the embedded charges, gate-dependent PL measurements were performed, as shown in Figure S23. The observed change in in *I*_A_–__/*I*_A0_ equivalent to the difference between Stack B and Control B corresponds to an effective *V*_gs_ of ∼60 V. Assuming full electrostatic coupling of the induced charges into the channel, this corresponds to a charge density of ∼4.5 × 10^12^ q cm^–2^, which is in close agreement with the embedded *Q*_surf_/*q* in Stack B.

Beyond differences arising from experimental methodology, electrostatic doping is subject to intrinsic physical limits imposed by interface states. As the Fermi level in MoS_2_ shifts, interface states can change their charge state, effectively screening the electric field without contributing mobile charge carriers in the channel. To evaluate the intrinsic limits of electrostatic doping imposed by interface states, we employ a self-consistent electrostatic model to provide a qualitative analysis. The Fermi level *E*_f_ is determined by enforcing charge neutrality between the embedded charge *Q*_surf_, the mobile channel carriers *Q*_channel_ and charged interface defects *Q*_it_

1 $$Q_{surf}+Q_{channel}\left(E_{f}\right)+Q_{it}\left(E_{f}\right)=0$$

For each *E*_f_, the channel electron and hole sheet densities were calculated using the Boltzmann approximation^62^

2 $$n=g_{s}g_{v}\frac{m_{e}^{*}k_{B}T}{2\pi \hbar^{2}}\exp\left(-\frac{E_{C}-E_{f}}{k_{B}T}\right),,p=g_{s}g_{v}\frac{m_{h}^{*}k_{B}T}{2\pi \hbar^{2}}\exp\left(-\frac{E_{f}-E_{V}}{k_{B}T}\right)$$

yielding the channel carrier density *Q*_channel_ = *q*(*n* – *p*). The charge contribution from donor-like and acceptor-like interface states is obtained by integrating their occupancies over energy

3 $$Q_{it}=q\int D_{it}\left(E\right)f\left(E,E_{f}\right)dE$$

The calculation is iterated until charge neutrality is satisfied, allowing the relationship between *Q*_surf_ and *Q*_channel_ to be quantified with a defined *D*_it_ distribution across the bandgap. A schematic illustrating the occupation of interface states and the resulting partitioning of the embedded charge between interface traps and the neighboring MoS_2_ channel is shown in Figure 5a. The interface state density *D*_it_(*E*) is parametrized by separating midgap (*D*_it,mid_) and band-edge (*D*_it,tail_) contributions, each comprising donor-like (*D*_it,mid/tail‑don_) and acceptor-like (*D*_it,mid/tail‑acc_) states, consistent with commonly reported interface state distributions in semiconductors.^63^ Further details of the calculation are included in Supporting Information.

**Figure 5:**
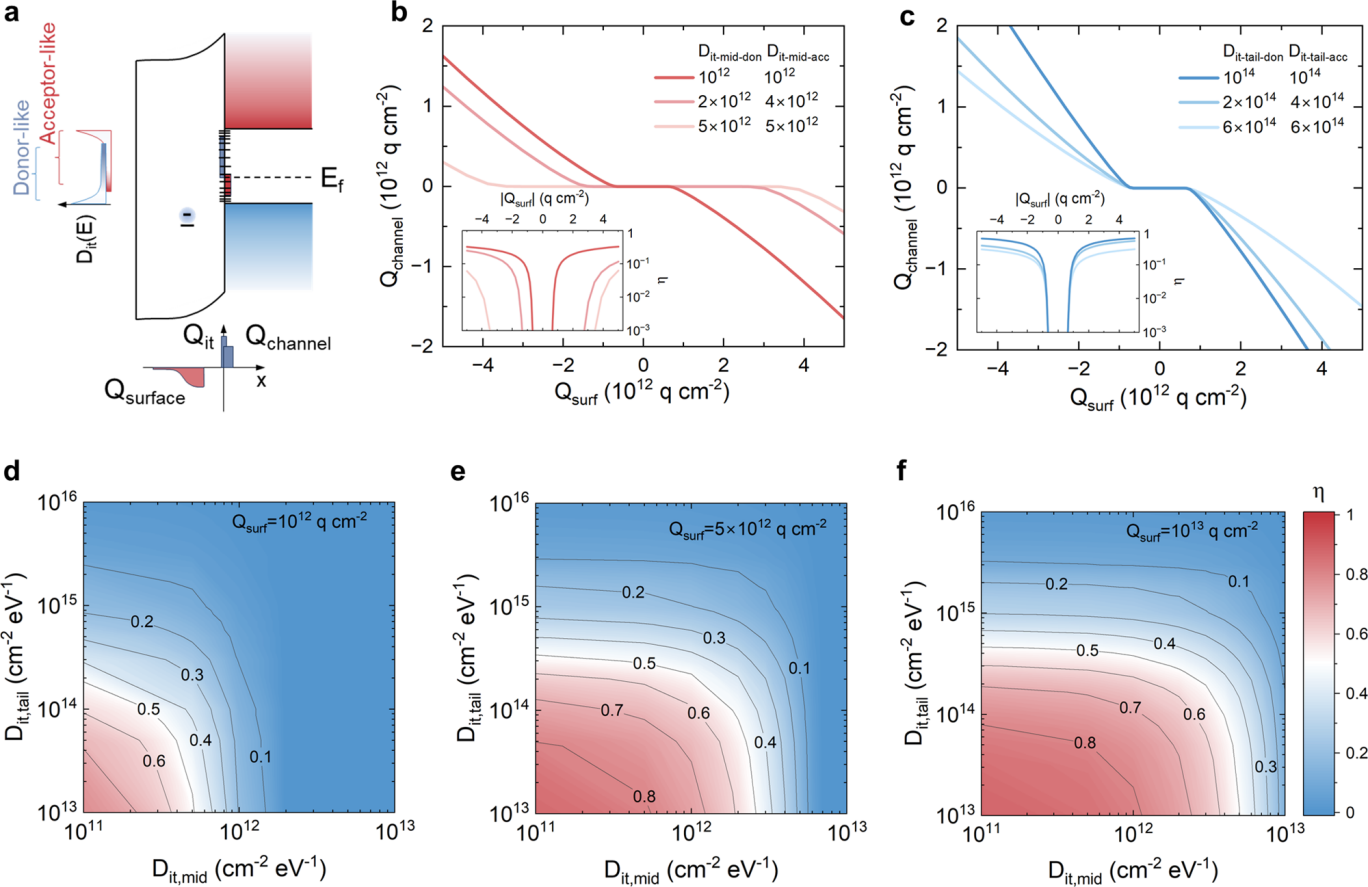
(a) Schematic band diagram illustrating the partitioning of embedded dielectric charge between occupied interface defect states and the MoS_2_ channel. The occupancy of defect states, set by the Fermi level *E*_f_, gives rise to an interface trap charge *Q*_it_ that screens part of the embedded surface charge, resulting in a reduced mobile channel charge *Q*_channel_ relative to *Q*_surf_. (b,c) Calculated mobile channel charge *Q*_channel_ as a function of embedded charge *Q*_surf_ for increasing (b) mid gap *D*_it,mid_ with *D*_it,tail_ = 5 × 10^14^ cm^–2^ eV^–1^ and (c) band-tail interface state densities *D*_it,tail_ with *D*_it,mid_ = 10^12^ cm^–2^ eV^–1^. Inset shows the coupling efficiency η = |*Q*_channel_|/|*Q*_surf_|. (d–f) Contour maps of η versus *D*_it,mid_ and *D*_it,tail_ with (d) *Q*_surf_ = 10^12^ q cm^–2^, (e) *Q*_surf_ = 5 × 10^12^ q cm^–2^, and (f) *Q*_surf_ = 10^13^ q cm^–2^.

The calculation reveals how interface states govern effective coupling of embedded dielectric charge and the 2D channel. Figure 5b and Figure 5c shows the calculated channel charge density *Q*_channel_ as a function of the embedded dielectric charge *Q*_surf_ for different *D*_it‑mid_ and *D*_it‑tail_. Two distinct regimes can be identified. At low |*Q*_surf_|, the channel charge density remains negligible despite increasing embedded charge, resulting in a coupling efficiency η = |*Q*_channel_|/|*Q*_surf_| ≪ 1. This regime corresponds to a dominant charge accumulation at the interface states. With further increase in |*Q*_surf_|, the interface states become progressively filled and additional charges are mirrored into the MoS_2_ channel, leading to a rapid increase in *Q*_channel_.

The extend of the low-coupling regime is primarily governed by defect states at midgap with higher *D*_it,mid_ leading to a wider “dead” region in which electrostatic doping is strongly suppressed. The low-coupling regime is also observed in gate-dependent PL spectra (Figure S23), providing experimental support for the model predictions. In contrast, band-tail states predominantly control the slope of *Q*_channel_ with increasing |*Q*_surf_|, with smaller *D*_it,tail_ yielding higher η. The coupling behavior can also be asymmetric between electron and hole doping, depending on the relative densities of donor- and acceptor-like states. Donor-like states primarily govern the coupling under negative *Q*_surf_ (*n*_el_ reduction), while acceptor-like states dominate under positive *Q*_surf_ (*n*_el_ increase).

To identify the conditions required for effective electrostatic coupling, Figure 5d–f present contour maps of the coupling efficiency η as a function of *D*_it,mid_ and *D*_it,tail_ at fixed values of *Q*_surf_/*q* = 1 × 10^12^, 5 × 10^12^, 1 × 10^13^ cm^–2^, with donor- and acceptor like state densities set equal. These maps show that achieving efficient electrostatic doping requires not only a sufficiently large embedded charge density, but also interface-state densities below critical thresholds. Within the present model, effective coupling (η > 0.5) is obtained only when *D*_it,mid_ ≲ 5 × 10^12^ cm^–2^ eV^–1^ and *D*_it,tail_ ≲ 5 × 10^14^ cm^–2^ eV^–1^ at *Q*_surf_/*q* of 10^13^ cm^–2^, highlighting the combined importance of charge density and interface quality on electrostatic doping.

To further extend the proposed electrostatic doping strategy toward CMOS-compatible circuit applications, several important challenges remain to be addressed. One key issue is the large-area uniformity of the embedded dielectric charge. For wafer-scale implementations, the spatial uniformity of the charge density is expected to depend primarily on (i) the uniformity of defect distributions within the dielectric layer and (ii) the uniformity of the charging process itself. In this work, the dielectric films are deposited by ALD, which is known for excellent conformality and large-area thickness uniformity due to its self-limiting growth mechanism. In addition, corona charging is a mature industrial process that has previously demonstrated good large-area charge uniformity.^64^

Another important challenge is achieving precise and controllable doping levels through accurate control of the injected dielectric charge. As shown in Figure 5, this additionally requires careful interface engineering to maintain strong electrostatic coupling between the embedded charge and the 2D channel. Long-term charge stability also requires further investigation. Finally, practical CMOS-level implementation will require the development of area-selective charging strategies compatible with device integration. While these aspects are beyond the scope of the present work, the results presented here establish a physical framework for future studies toward scalable electrostatic doping in 2D electronics.

## Conclusions

We demonstrate an electrostatic doping strategy for monolayer MoS_2_ based on embedded fixed charge within engineered dielectric stacks. Using simple corona charging-based processes under ambient conditions, negative charge can be introduced several nanometres under the dielectric surface, generating a built-in electrostatic field that modulates the carrier density in the adjacent 2D channel without requiring continuous external bias or compromising carrier mobility.

By comparing multiple charged dielectric architectures, we find that the effectiveness of electrostatic doping depends sensitively on defect states in the capping dielectric and its interface with the 2D channel. No electrostatic doping is observed in samples where the charging process introduces a high density of defects in the capping dielectric. In contrast, embedding charge prior to deposition of a low-defect HfO_*x*_ capping layer enables electrostatic doping of 2D MoS_2_ demonstrated by both transport measurements and PL spectroscopy.

A self-consistent electrostatic model further reveals that defect states at the dielectric-2D channel interface intrinsically constrain the efficiency of electrostatic doping. Midgap states define the onset of effective coupling between the embedded charge and the 2D channel, while band-tail states control the efficiency of additional embedded charges are mirrored into the 2D channel. Together, these results establish quantitative design criteria for the charged dielectric and its interface with the channel required for effective electrostatic doping in two-dimensional semiconductors.

This work highlights the broad scope of dielectric defect engineering in controlling the electronic properties of two-dimensional semiconductors. By shifting the focus of doping strategies from the 2D channel to the surrounding dielectric, carrier modulation can be achieved without direct chemical or structural modification of the 2D channel. Dielectric defect engineering thus offers a versatile platform for electrostatic modulation in 2D semiconductors.

## Methods

### Substrate Preparation and Dielectric Charging

Silicon wafers with 300 nm wet thermal SiO_2_ were used as substrates. For Stack A and Control A, AlO_*x*_ and SiO_*x*_ layers were deposited sequentially by ALD, consisting of 20 cycles of AlO_*x*_ (∼2 nm) followed by 360 cycles of SiO_*x*_. The samples were annealed at 800 °C in air for 30 min prior to charging to reduce dielectric bulk defect density. For Stack B, the substrates were first annealed at 800 °C in air for 30 min, followed by hot corona charging and subsequent deposition of a 50-cycle HfO_*x*_ (∼5 nm) capping layer by ALD. Further details of the film thickness measurement and ALD recipe are included in Supporting Information.

Corona charging at room temperature was performed by placing the sample on a grounded copper plate beneath a metal tip electrode with a tip-plate separation of 20 cm, while a voltage of ±30 kV was applied to the tip electrode. For hot corona charging, an aluminum plate on a hot plate was used as the ground electrode placed on a hot plate in air. The tip-plate separation was 16 cm and the tip voltage was set to −20 kV. Control A and B samples underwent identical thermal treatments without corona charging. Stack A samples used for studying doping of 2D MoS_2_ were subjected to a repeated corona-anneal process, consisting of 30 s of negative corona charging followed by annealing at 450 °C for 1 min in air, repeated four times. Stack B samples were charged at 450 °C for 5 min in air prior to HfO_*x*_ deposition.

Surface charge density was characterized using a scanning Kelvin probe (SKP5050, KP Technology) equipped with a 2 mm diameter Au probe. The effective surface charge density was extracted from the measured contact potential difference (CPD) assuming a charge centroid located near the SiO_2_ surface (*x*_c_ = 300 nm), according to

4 $$V_{b}=-CPD=-\left(\frac{\Phi_{m}-\Phi_{s}}{q}-\frac{Q_{surf}x_{c}}{\varepsilon_{diel}}-\phi_{scr}\right)$$

where ϕ_m_ and ϕ_s_ are the probe and silicon work functions, respectively, ϕ_scr_ is the surface potential arising from the space charge region, which is negligible in the present work,^35^ and ε_diel_ is the dielectric permittivity.

### Device Fabrication

Monolayer MoS_2_ flakes were mechanically exfoliated from bulk crystals using adhesive tape and transferred onto the target substrates using polydimethylsiloxane (PDMS) stamps. The number of layers was first identified by optical contrast under an optical microscope. Representative samples were subsequently characterized by Raman spectroscopy to confirm the correspondence between optical contrast and layer thickness. Details of the Raman spectra are included in Supporting Information. Monolayer flakes with lateral dimensions of approximately 10–20 μm were selected. Aluminum contacts were deposited by thermal evaporation to form the source and drain electrodes using transmission electron microscopy (TEM) grids as shadow masks, while a full area aluminum layer was evaporated onto the silicon as the back gate electrode. While the devices exhibit contact-limited transport due to nonoptimized Al contacts, resulting in low turn-on currents, this limitation is not expected to affect the relative trends in carrier modulation. No wet chemical processing was employed during fabrication to preserve the surface charge embedded in the dielectric.

To demonstrate electrostatic carrier modulation using a polymer dielectric, FET devices with a PMMA capping layer were fabricated on a Control B substrate. PMMA was spin-coated onto completed devices and annealed at 180 °C for 90 s. Positive or negative corona charge was deposited at room temperature for 20 s each step.

### Electrical Characterization

Electrical transport measurements were performed in the dark under a flowing argon atmosphere. After contacting the device inside an enclosed measurement chamber, the chamber was purged with argon for 20 min prior to measurement to minimize ambient exposure. Transfer characteristics were measured by sweeping the gate voltage from 0 to 80 V and back to 0 V in 2 V steps to capture hysteresis. For Stack B devices, the gate voltage range was extended to 120 V to fully capture channel turn-on. Five consecutive sweeps were recorded for each device to allow the transport characteristics to stabilize, with the response converging by the final sweep. Threshold voltage (*V*_th_) was extracted by linear extrapolation of the transfer curves between 70 and 80 V to ensure comparison across devices on multiple substrates. Field-effect mobility μ_FE_ was obtained from the slope of the linear regime of the forward transfer characteristics of the full measured gate voltage range, and hysteresis was quantified from the difference in *V*_th_ between forward and reverse sweeps. The drain-source voltage was applied using a Keithley 2401 source meter, and the gate-source voltage was applied using a Keithley 487. All raw transport data are provided in the Supporting Information.

### Photoluminescence Spectroscopy

PL measurements were performed at room temperature in air using a confocal Raman microscope (Horiba LabRAM Aramis) with a 532 nm excitation laser. Each spectrum was averaged over five acquisitions with an integration time of 3 s. The PL spectra were fitted using Lorentzian functions to extract the integrated intensities of the neutral exciton (A^0^) and negatively charged trion (A^–^) peaks.^65^ The ratio in *I*_A_–__/*I*_A0_ was used to estimate for local electron density monolayer MoS_2_ based on a mass-action model describing the equilibrium between A^0^, A^–^ and free electrons.^66^ Relevant model parameters were taken from the literature and are detailed in Supporting Information.

## Supplementary Material



## Data Availability

The data that support the findings of this study are available from the corresponding author upon reasonable request.

## Notes

Details of corona charging and dielectric fabrication; surface charge extraction from Kelvin probe measurements; supplementary transport and photoluminescence data; optimization of dielectric thickness; additional control experiments; electrostatic modeling details (PDF)
