# Supplementary material for: Electrostatic Doping of 2D Semiconductors Using Charged Dielectric Thin Films
Source: ACS Nano. 2026 Jul 15;20(29):20787–97. doi: 10.1021/acsnano.6c07356 (PMC13421956; doi:10.1021/acsnano.6c07356)
Supplement: Supplementary file 1 [file nn6c07356_si_001.pdf]

Supporting Information for Electrostatic Doping of 2D Semiconductors using Charged Dielectric Thin Films

*Xinya Niu[a,b,c], John O'Sullivan[a], Bin Han[d], Lixin Liu[e], Yi Cui[c], Yan Wang[e], Manish Chhowalla[e], Ruy S. Bonilla[a,b]**

[a] Department of Materials, University of Oxford, Oxford, OX1 3PH, United Kingdom
[b] Oxford Suzhou Centre for Advanced Research (OSCAR), University of Oxford, Suzhou 215123, China
[c] Suzhou Institute of Nano-tech and Nano-bionics, Chinese Academy of Sciences, Suzhou 215123, China
[d] University of Strasbourg, CNRS, ISIS UMR 7006, Strasbourg, F-67000, France
[e] Department of Materials Science & Metallurgy, University of Cambridge, Cambridge, CB3 0FS, United Kingdom

*Corresponding author: sebastian.bonilla@materials.ox.ac.uk

Supporting Data for Doping from a Charged PMMA Top Dielectric

Table S 1 Extracted threshold voltage (V_{th}), measured contact potential difference (CPD) from Kelvin probe measurement on charged PMMA on a MoS_2 field-effect transistor (FET), and their corresponding channel electron density (n_{el}) and surface charge density (Q_{surf}) with increasing accumulated charging time. Between 0-80 seconds, negative corona charges are deposited, and between 100-140 seconds, positive corona charges are deposited.

Accumulated Charging Time (s)	V_{th} (V)	n_{el} (10^{11} q cm^{-2})	CPD (V)	Q_{surf} (10^{12} q cm^{-2})
0	-4.25	3.05	-1.26	0.03
20	1.43	-1.03	24.55	-1.08
40	6.26	-4.51	43.88	-1.91
60	9.20	-6.62	52.80	-2.30
80	9.19	-6.61	49.03	-2.14
100	7.99	-5.75	9.09	-0.41
120	3.93	-2.83	-13.86	0.58
140	-0.91	0.65	-32.35	1.37

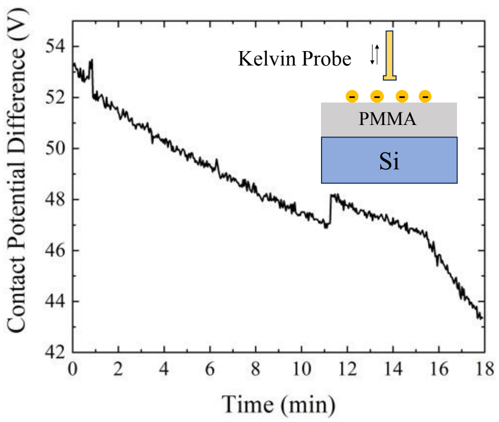

Figure S 1 Degradation of the CPD values of negative corona charge on PMMA. The abrupt change in CPD value is likely due to a small vibration of the instrument during the long measurement, as CPD value is very sensitive to the sample-probe distance.

Supporting Data for Design and Fabrication of Negatively Charged Oxide Nanolayers

• Capping Layer Thickness Optimisation for Stack A

The SiO_x capping layer thickness was optimised by analysing temperature-dependent charge trapping at the SiO_x/AlO_x interface. The aim was to identify a thickness range that enables electron injection at elevated temperatures while suppressing charge transfer at lower temperatures. Silicon was used as the substrate to provide an electron source for charging interface defects.

A SiO_x layer (~500 ALD cycles) was deposited on a 1 × 7 cm Si substrate and annealed at 800 °C for 30 min. A thickness gradient was created by controlled HF etching, and the local SiO_x thickness was determined by ellipsometry. Subsequently, 20 cycles each of AlO_x and SiO_x were deposited by ALD. The samples were annealed at temperatures between 150 and 450 °C for 10 min per step. After each anneal, Kelvin probe measurements were performed under dark and illuminated conditions, and the surface photovoltage ($SPV=CPD_{illuminated}-CPD_{dark}$) was extracted as a function of SiO_x thickness.

The SPV profiles exhibit three regimes: low-SPV, transition, and high-SPV regions. Thin SiO_x layers show negative SPV values due to efficient electron injection and charge trapping at the SiO_x/AlO_x interface, with higher annealing temperatures yielding increased negative charge density. With increasing SiO_x thickness, SPV decreases as electron injection becomes progressively suppressed. For sufficiently thick SiO_x layers, SPV stabilises at positive values, indicating minimal electron injection and dominant positive charge near the Si/SiO_x interface.[1]

Based on these observations, the optimal SiO_x thickness is identified as the overlapping range that allows charge injection at 450 °C while preventing charge transfer at temperatures ≤150 °C, corresponding to approximately 10–26 nm.

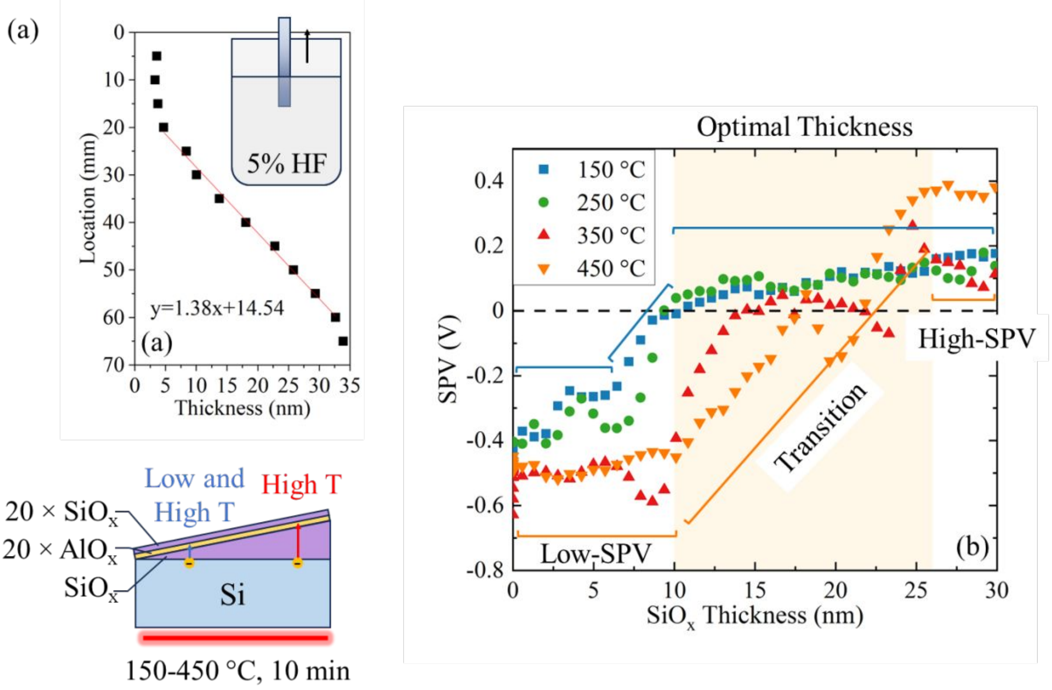

Figure S 2(a) measured SiO_x thickness along the etching direction, and (b) SPV profile measured as a function of SiO_x thickness after annealing at 150-450 °C for 10 minutes at each temperature. The blue and orange brackets mark the low-SPV, transition and high-SPV regions of the SPV profiles obtained after annealing at 150 and 450 °C, respectively. Each point represents the average of 50 measurements at a single location.

• Corona Charge Stability under Different Temperatures

Negative corona charge was deposited for 90 s on Si substrates with 300 nm SiO_2 and subsequently annealed at different temperatures. Charge dissipation accelerated with increasing annealing temperature, suggesting increased de-trapping at elevated temperatures and explaining the enhanced charge density obtained under hot corona charging at lower temperatures.

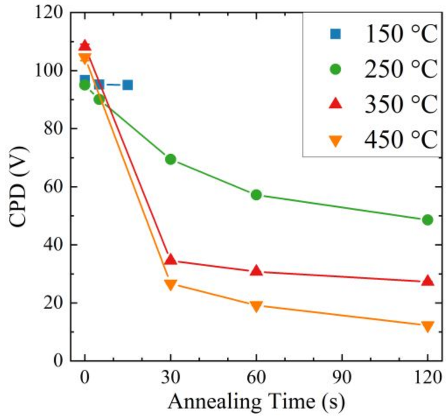

Figure S 3 CPD values with increasing annealing time under different temperatures between 150-450 °C. All samples (Si substrate with 300 nm SiO_2) were deposited with 90 seconds of negative corona charge at room temperature prior to annealing.

• Charge Retention after ALD Process

To evaluate charge retention in ALD-HfO_x, Si substrates with 300 nm SiO2 were annealed at 800 °C for 30 min and hot corona-charged at 450 °C for 2–10 min. Subsequently, 50 ALD cycles of either SiO_x or HfO_x were deposited using ALD. Surface charge densities before and after deposition were measured by Kelvin probe. Compared to SiO_x-capped samples, HfO_x-capped samples exhibited reduced negative charge loss, indicating improved charge preservation. Charge reduction observed for both capping layers is attributed to exposure to ALD precursor gases during the initial deposition cycles.

Table S 2 Measured charge densities on hot corona-charged samples before and after depositing 50 cycles of SiO_x or HfO_x.

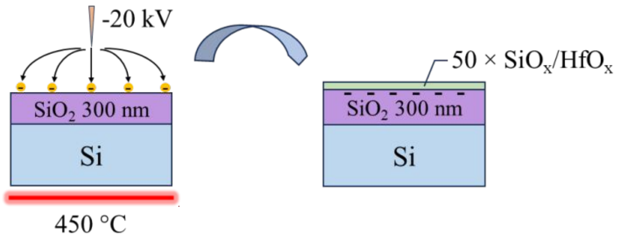

Capping Dielectric	Charging Time (min)	Charge Density (10^{12} q cm^{-2}) Before ALD	After ALD	Change (%)
SiO_x	2	-2.5	-1.1	-56
	5	-6.6	-2.8	-58
HfO_x	2	-2.3	-2.0	-13
	5	-5.4	-4.8	-11
	8	-9.3	-9.3	0
	10	-8.5	-8.1	-5

• Charge Retention of 5 nm HfO_x Films

To assess the suitable HfO_x thickness to prohibit charge degradation, a ~500-cycle HfO_x film with a thickness gradient was deposited on a 1 × 7 cm^2 Si substrate by ALD. Following deposition of 20 ALD cycles each of AlO_x and SiO_x to form a SiO_x/AlO_x interface, the samples were annealed between 150 and 450 °C for 10 min per step. SPV and dark CPD profiles were measured as functions of HfO_x thickness.

Across all annealing temperatures, the SPV response exhibits a clear transition at an HfO_x thickness of approximately 5 nm. Below this thickness, reduced charge retention is observed, whereas for HfO_x layers ≥ ~5 nm, the SPV and CPD signals stabilise, indicating effective suppression of electron injection from the Si substrate. This thickness-independent behaviour beyond ~5 nm demonstrates that a thin HfO_x layer is sufficient to retain embedded charge and electrically isolate the SiO_x/AlO_x interface. The observed temperature-dependent polarity of the charge is attributed to annealing-induced structural changes at the Si/HfO_x interface, consistent with previous reports.[2,3]

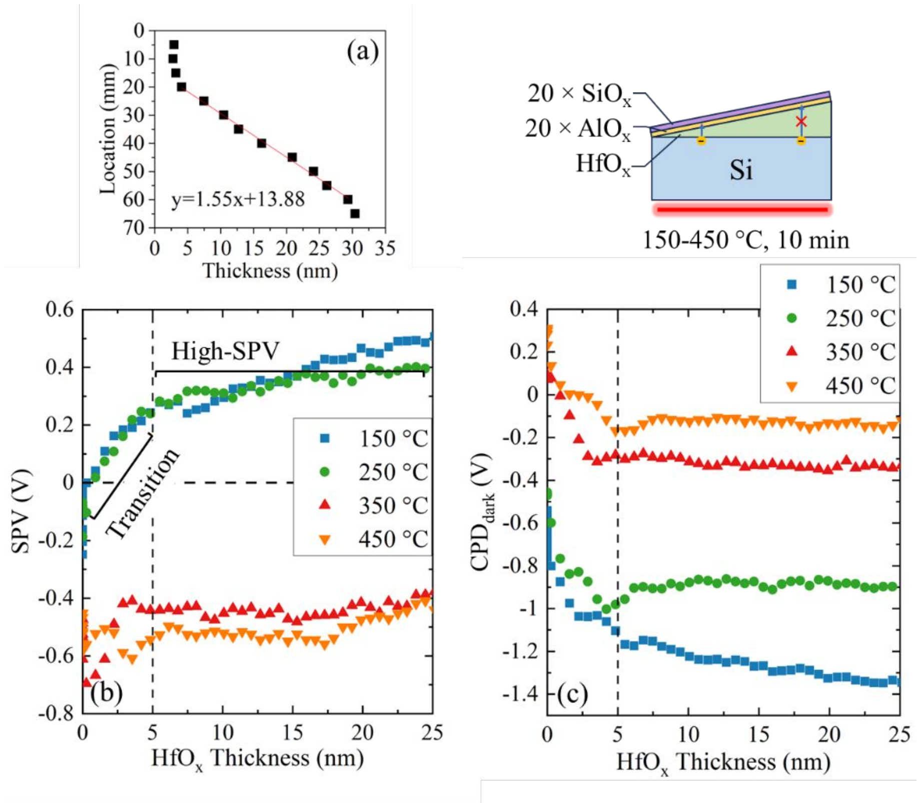

Figure S 4(a) Thickness measured across the sample. (b) SPV profiles and (c) CPD profiles measured in dark as a function of HfO_x thickness, measured after annealing at different temperatures. The black brackets mark the transition and high-SPV region for SPV profiles obtained after annealing at 150-450 °C. Each point represents the average of 50 measurements at a single location.

• Additional Data on Charging Sequence

A dielectric stack consisting of 10 cycles of AlO_x and 50 cycles of HfO_x was deposited on a Si substrate with 300 nm thermal SiO_2 to evaluate charge injection through HfO_x. A corona-anneal charging method resulted in a rapid surface charge decay, indicating suppressed charge injection. After hot corona charging at 450 °C for 5 min and DI water rinsing, the same charging sequence showed improved charge retention, exceeding that of an AlO_x/SiO_x stack without hot corona charging. These results indicate that hot corona charging modifies the defect landscape within the dielectric stack, forming a charge injection/degradation pathway in the HfO_x layer.

To compare the charge retention characteristics of SiO_x, AlO_x and HfO_x, 100 cycles of AlO_x was deposited on a Si substrate with 300 nm thermal SiO_2, forming a SiO_x/AlO_x interface. The sample was then annealed at 450 °C for 5 minutes, followed by a corona-annealing test, as shown below. Compared with SiO_x- and HfO_x-capped samples, the AlO_x-capped structure preserved a larger fraction of the embedded charge. Since charging and discharging processes are intrinsically coupled, these results also reflect the relative charge-retention capabilities of the different dielectric systems.

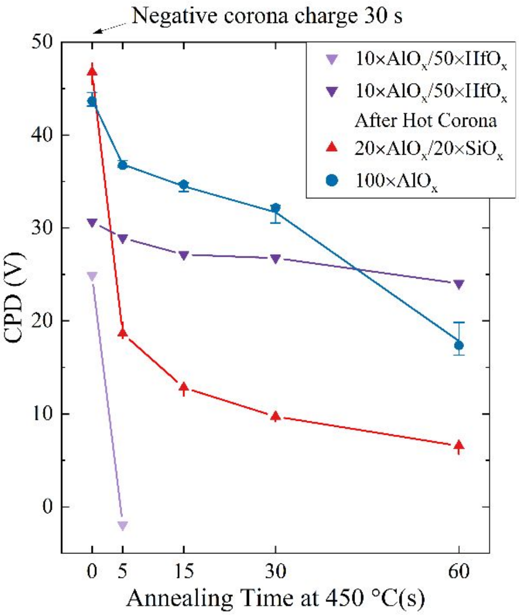

Figure S 5 CPD values with increasing annealing time at 450 °C after 30-seconds of negative corona charge deposition at room temperature. The different symbols represent different ALD layers and previous charging procedures.

• Additional Information on ALD Process and Film Thickness Measurement

Thin film dielectrics used in this work were deposited using a Vecco Savannah 200. Details of the process are provided in the table below:

Table S 3 Measured charge densities on hot corona-charged samples before and after depositing 50 cycles of SiO_x or HfO_x.

		SiO_x	AlO_x	HfO_x
Chamber Temperature (ºC)		150	150	150
Base Pressure (mTorr)			250	
N_2 Flow (sccm)		20	20	20
Precursor	Name	BDEAS	TMA	TDMAHf
	Precursor Temperature (ºC)	50	-	75
	Pulse Time (s)	0.10	0.015	0.15
	Purge Time (s)	5	8	20
Co-reactant	Name	O_3	H_2O	H_2O
	Pulse Time (s)	0.075	0.015	0.015
	Exposure Time (s)	20	-	-
	Purge Time (s)	5	8	20

The deposited capping SiO_x layer thickness in Control A and Stack A is measured to be 28 nm using ellipsometry prior to the 800 °C annealing, while the targeted ALD-SiO_x thickness is approximately 24.5 nm (360 cycles), determined from the optimisation study shown in Figure S2. The ALD cycle count was estimated using the apparent deposition rate extracted from the unetched region of the gradient etched sample (Figure S 2a). However, this likely underestimated the true growth rate due to partial reduction of the "unetched" SiO_x thickness during HF vapour exposure. In addition, the reported 28 nm thickness was measured on an as-deposited film on a dummy Si substrate prior to the 800 °C anneal, whereas the optimisation study was performed after annealing, which may further reduce the film thickness through densification. Film thickness reduction of ~10% have been reported for ALD-

SiO_x upon 900 °C annealing.[4] The remaining discrepancy is within the experimental uncertainty of ellipsometry thickness extraction and Kelvin probe measurements.

Supporting Data for Thermionic Emission and Tunnelling Probability

A simplified calculation is provided here to support the proposed defect-mediated charge-relaxation mechanism. The thermionic emission probability follows an Arrhenius-type dependence:

$$P \propto exp(-\frac{\Phi}{k_B T})$$

Assuming the charged defects lie near the middle of the SiO_2 bandgap, the estimated thermal emission barriers are $\Phi > 4.5$eV for Stack A and $\Phi > 2.65$eV for Stack B (defined by the energy difference between the HfO_x conduction band and the SiO_2 midgap defect level). The calculated dependence of P on $\Phi$is shown below. The emission probability decreases exponentially with increasing barrier height and becomes negligibly small within the relevant energy range considered here, suggesting that thermionic emission is unlikely to be the dominant charge-relaxation mechanism.

The direct tunnelling probability can be estimated from WKB form:

$$T \approx exp(-2\kappa d), \qquad \kappa = \sqrt{\frac{2m^*\Phi}{\hbar^2}}$$

Where a rapid decrease in the direct tunnelling probability is found for both Stack A and Stack B.

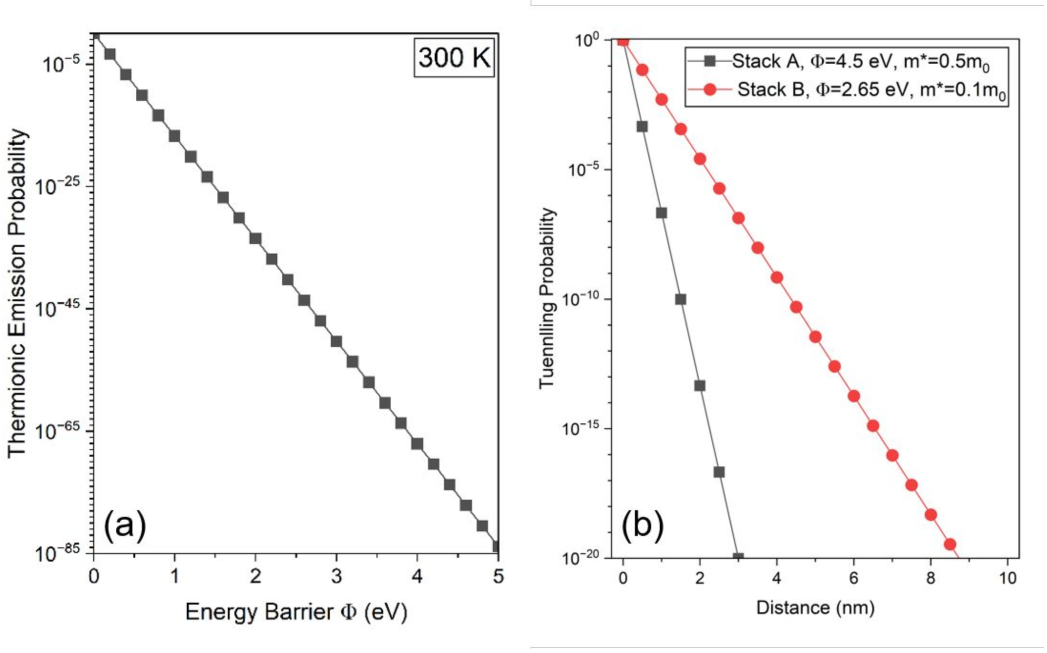

Figure S 6 Calculated (a) thermionic emission probability as a function of energy barrier Φ and (b) direct tunnelling probability as a function of distance.

Supporting Data for Electrostatic Doping of MoS_2 via Charged Dielectric Nanolayers

- Channel Optical Images, Transfer Curves, and Substrate Charge Density

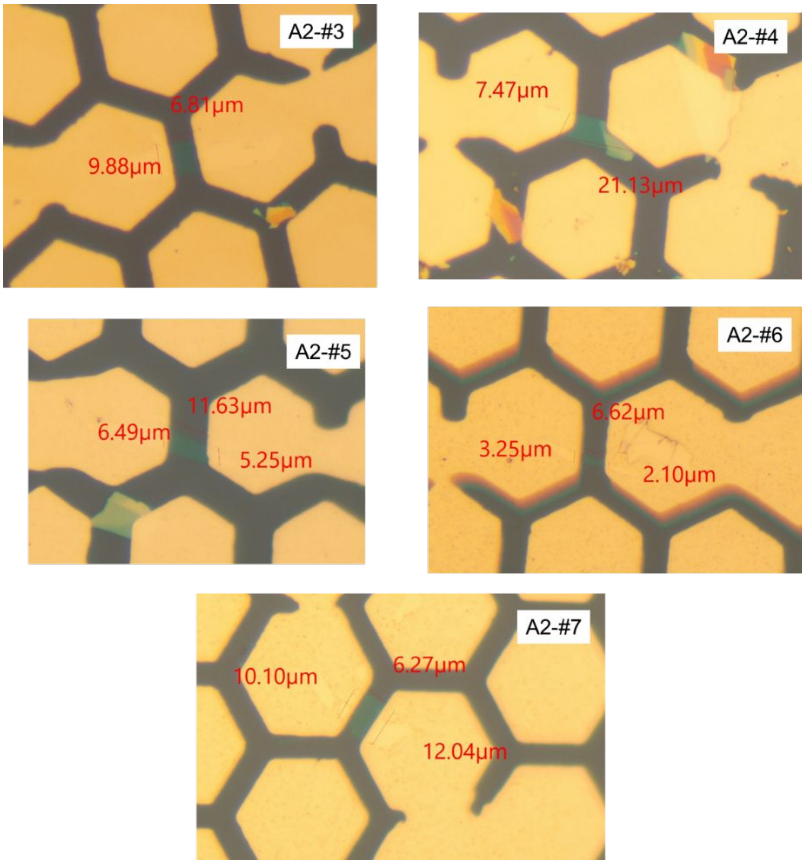

Figure S 7 Optical images of monolayer MoS_2 field-effect transistors on Control A.

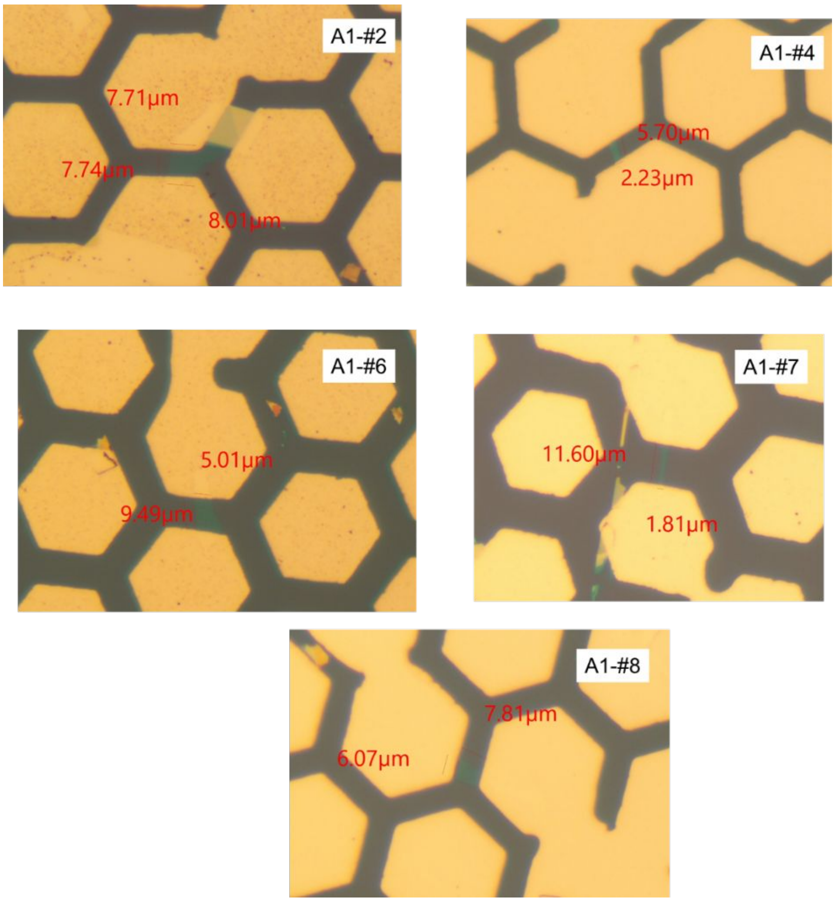

Figure S 8 Optical images of monolayer MoS_2 field-effect transistors on Stack A.

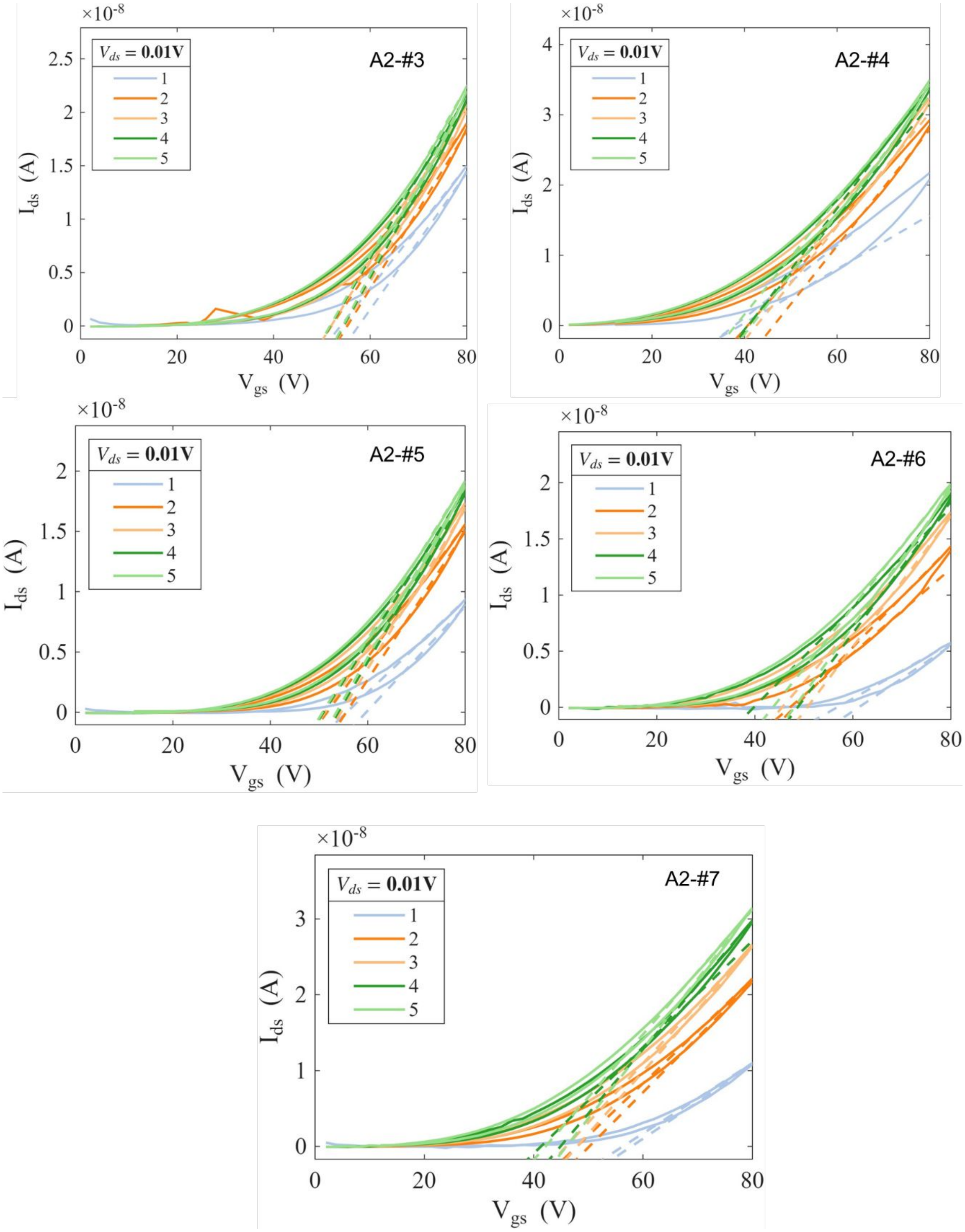

Figure S 9 Five transfer curves measured on each device on Control A substrates. Measurements were carried out in Ar environment in dark. Transfer curves demonstrate converging features by the fifth measurement, indicating device stabilisation.

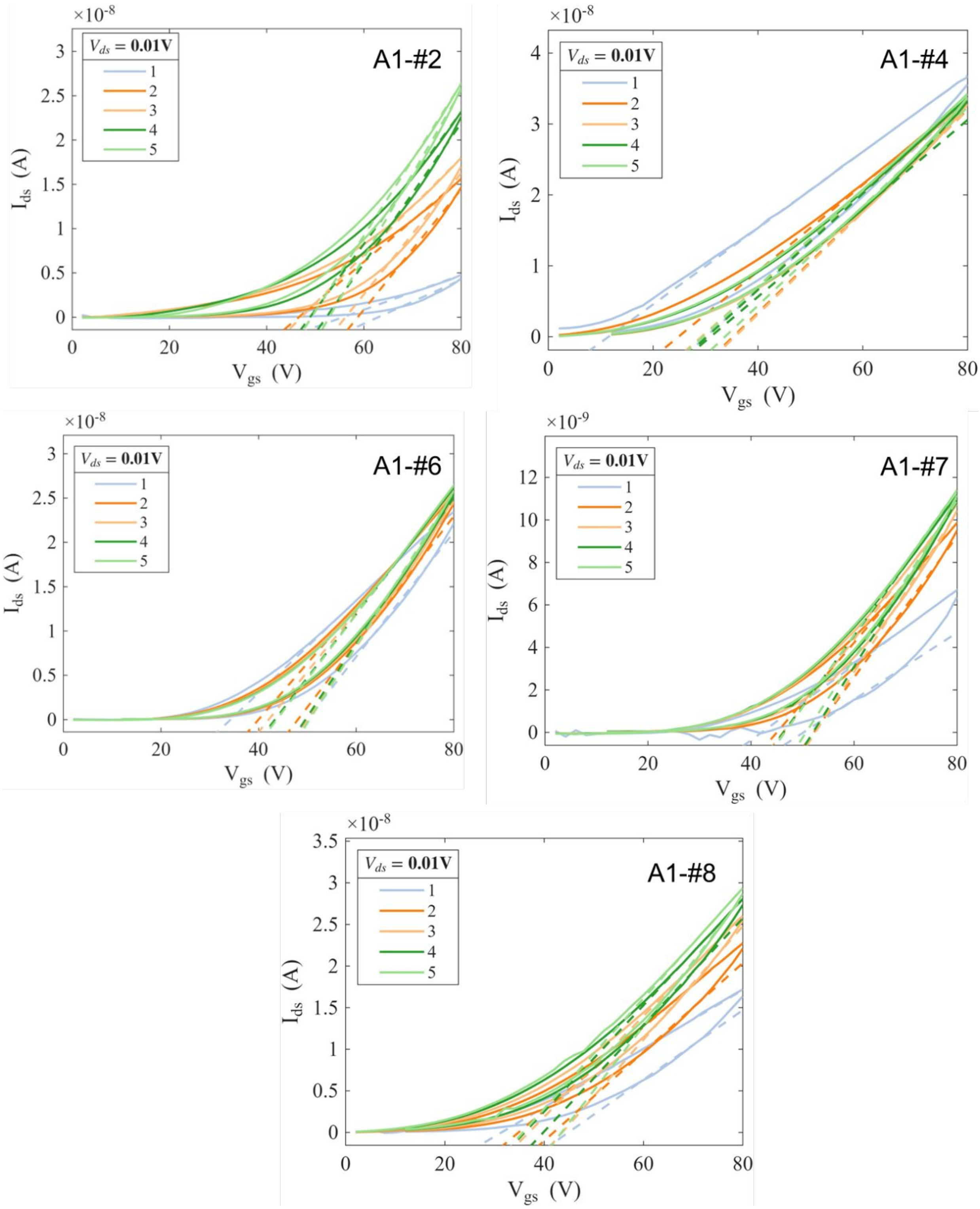

Figure S 10 Five transfer curves measured on each device on Stack A substrates. Measurements were carried out in Ar environment in dark. Transfer curves demonstrate converging features by the fifth measurement, indicating device stabilisation.

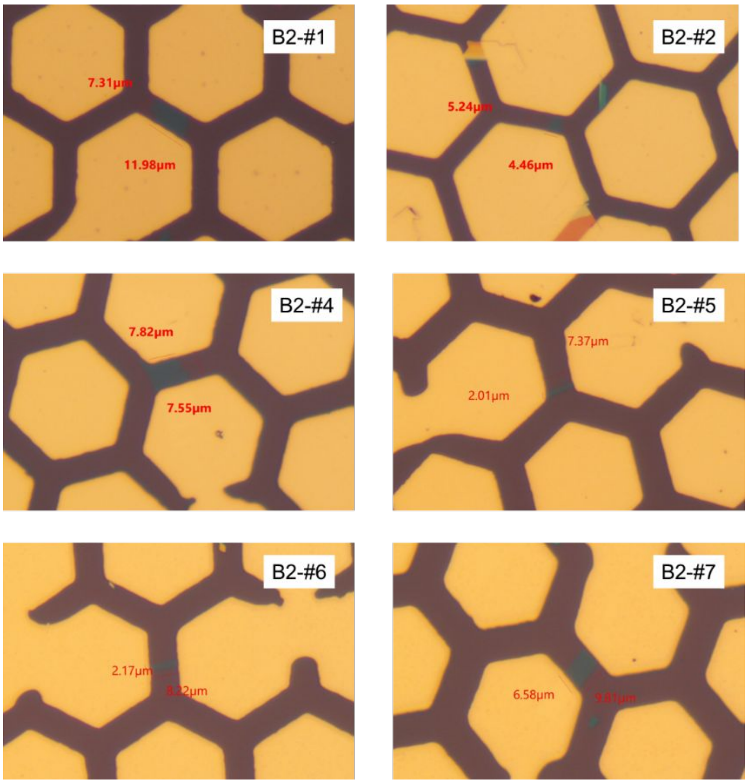

Figure S 11 Optical images of monolayer MoS_2 field-effect transistors on Control B.

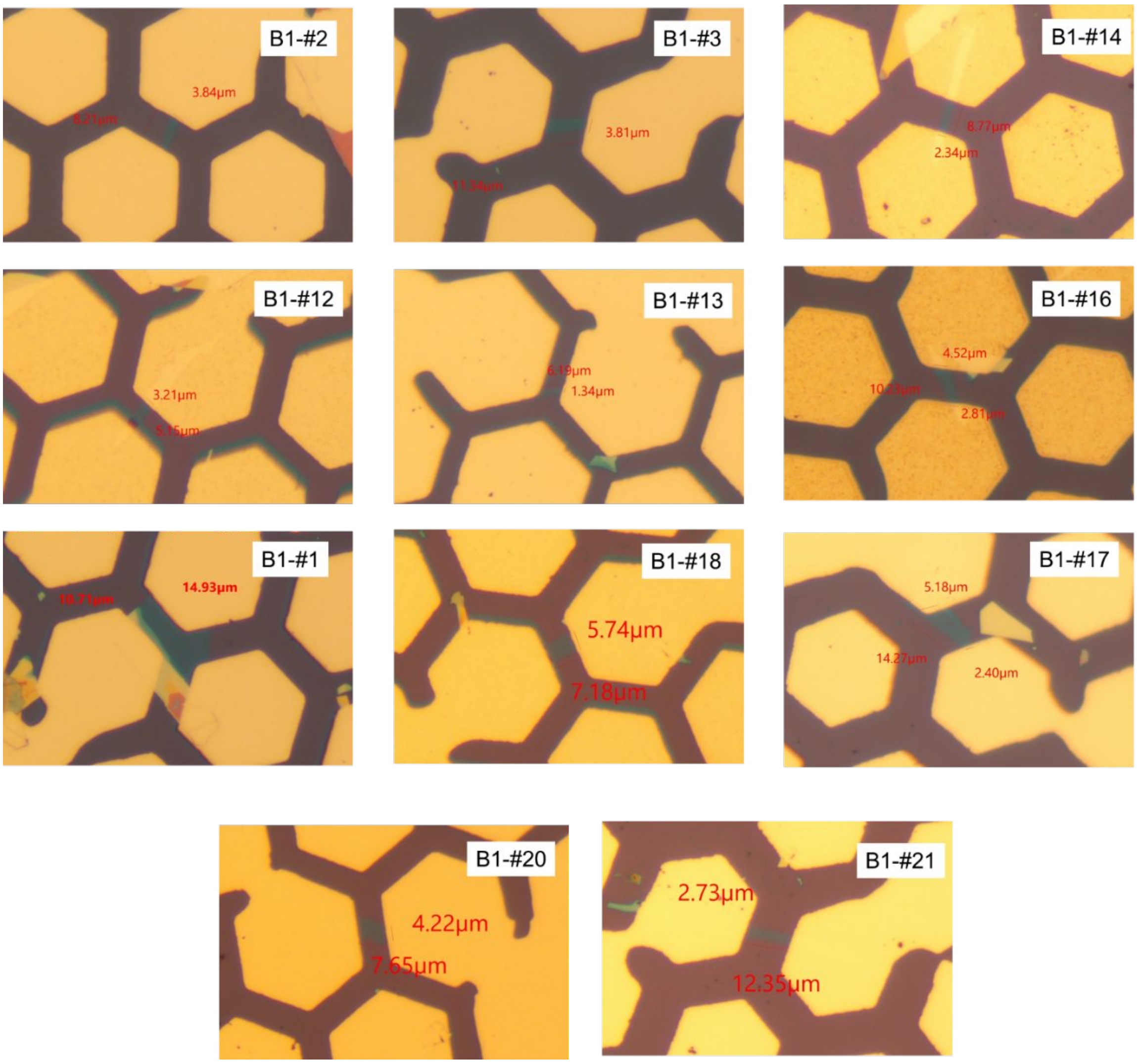

Figure S 12 Optical images of monolayer MoS_2 field-effect transistors on Stack B.

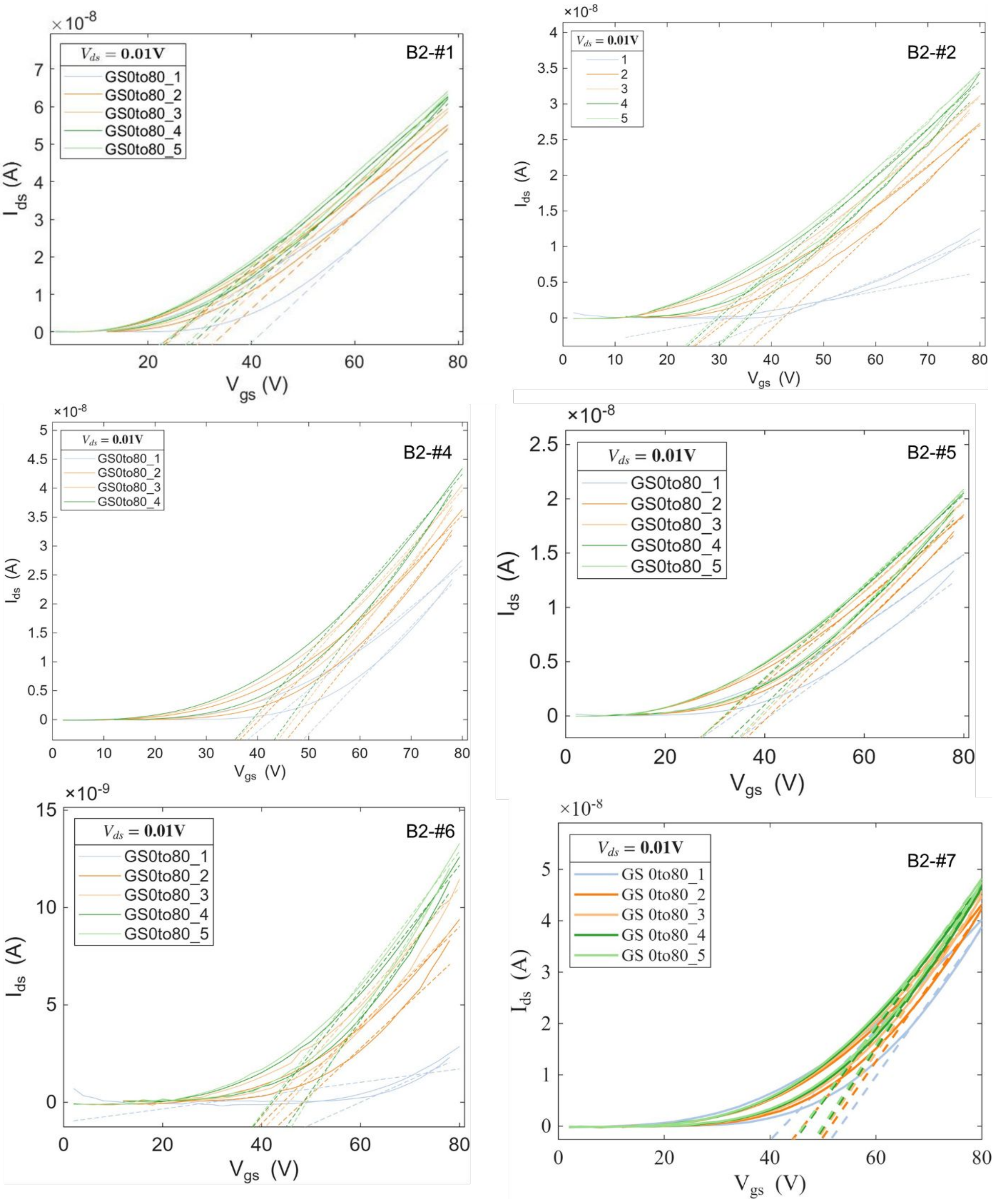

Figure S 13 Five transfer curves measured on each device on Control B substrates. Measurements were carried out in Ar environment in dark. Transfer curves demonstrate converging features by the fifth measurement, indicating device stabilisation.

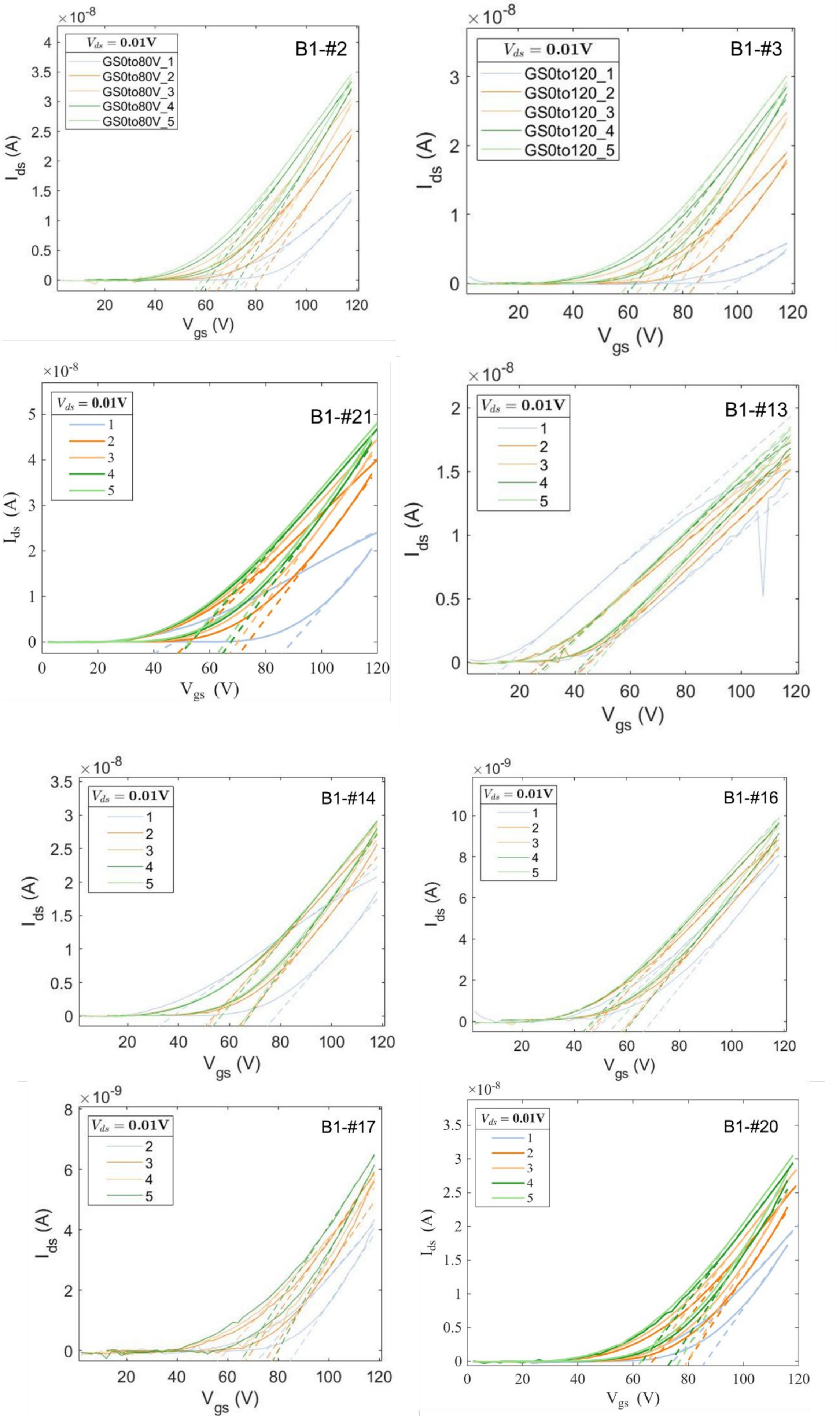
B1-#2
$V_{ds} = 0.01V$
GS0to80V_1
GS0to80V_2
GS0to80V_3
GS0to80V_4
GS0to80V_5
×10^-8
I_{ds} (A)
V_{gs} (V)
B1-#3
$V_{ds} = 0.01V$
GS0to120_1
GS0to120_2
GS0to120_3
GS0to120_4
GS0to120_5
×10^-8
I_{ds} (A)
V_{gs} (V)
B1-#21
$V_{ds} = 0.01V$
×10^-8
I_{ds} (A)
V_{gs} (V)
B1-#13
$V_{ds} = 0.01V$
×10^-8
I_{ds} (A)
V_{gs} (V)
B1-#14
$V_{ds} = 0.01V$
×10^-8
I_{ds} (A)
V_{gs} (V)
B1-#16
$V_{ds} = 0.01V$
×10^-9
I_{ds} (A)
V_{gs} (V)
B1-#17
$V_{ds} = 0.01V$
×10^-9
I_{ds} (A)
V_{gs} (V)
B1-#20
$V_{ds} = 0.01V$
×10^-8
I_{ds} (A)
V_{gs} (V)

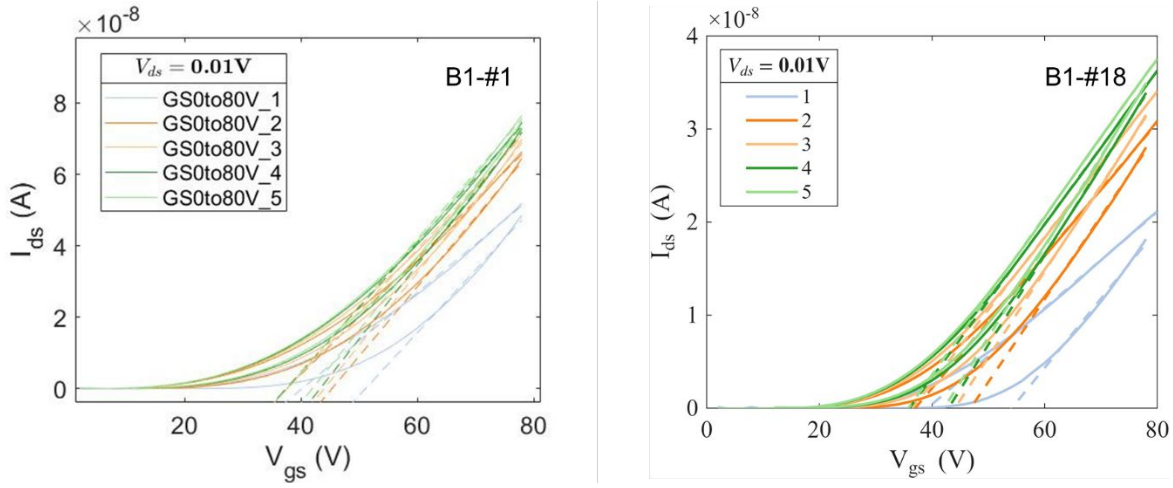

Figure S 14 Five transfer curves measured on each device on Stack B substrates. Measurements were carried out in Ar environment in dark. Transfer curves demonstrate converging features by the fifth measurement, indicating device stabilisation.

- Additional transfer characteristics on log-scale

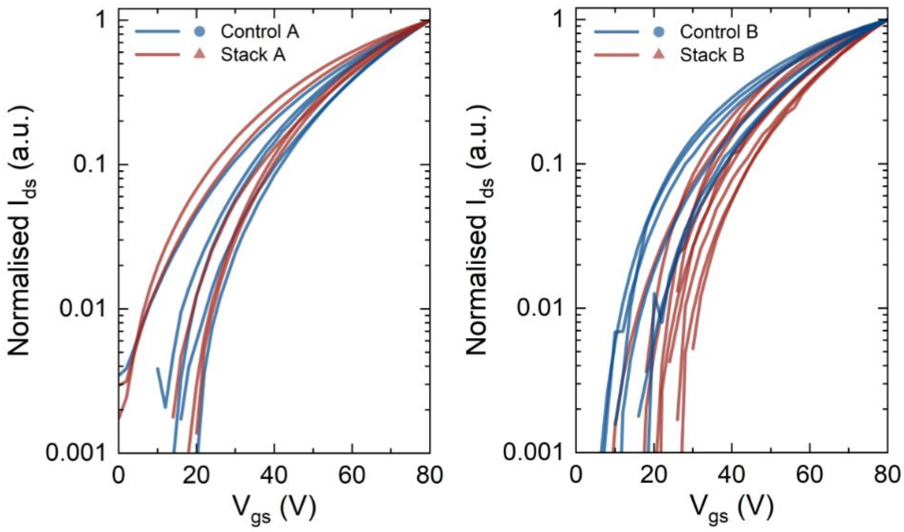

Figure S 15 Transfer curves plotted on a logarithmic scale to highlight the subthreshold turn-on behaviour. The absence of a well-defined turn-on point suggests non-ideal contact interface quality and relatively high contact resistance, which may also contribute to the less pronounced shifts in threshold voltage.

Substrate charge density variations were characterised by Kelvin probe scanning. To minimise the influence of surface contamination, charge uniformity was evaluated only once on the hot corona-charged substrate prior to sample cutting. All substrates were initially charged as 3×3 cm^2 samples and subsequently diced into 1×1 cm^2 pieces for device fabrication. The observed non-uniformity is primarily attributed to the corona charging configuration, including the probe geometry, probe-sample separation, and sample dimensions.[5] As these parameters were kept constant throughout this study, the measured non-uniformity is considered representative of the charge distribution across all samples.

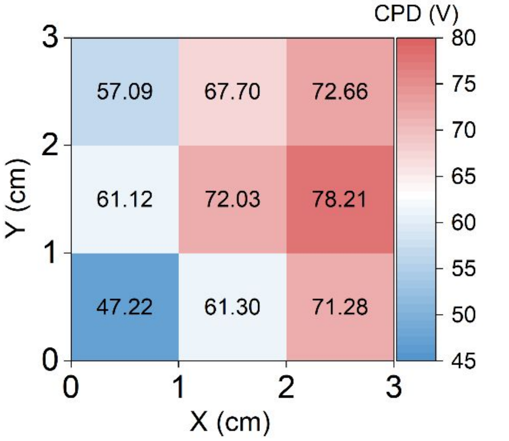

Figure S 16 Contact potential difference measured on a 3×3 cm^2 substrate charged for 5 minutes using the hot corona method at 450 °C. The substrate is later cut into 1×1 cm^2 samples to make Stack B devices. The same process was used to make all substrates for device fabrication.

- Supplementary Raman Spectra

Raman spectra on different substrates are provided here to confirm the monolayer selection through optical microscopy.[6]

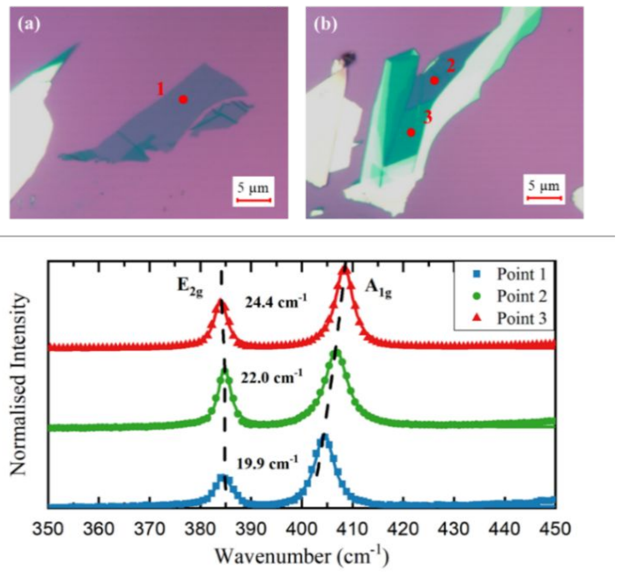

Figure S 17 Exemplary optical microscope photos of exfoliated (a) monolayer and (b) multilayer flakes and their corresponding (c) Raman spectrums, which were obtained at the location indicated in (a) and (b) with red dots. Exfoliated flakes are transferred onto Si substrates with a 300 nm SiO_2 layer.

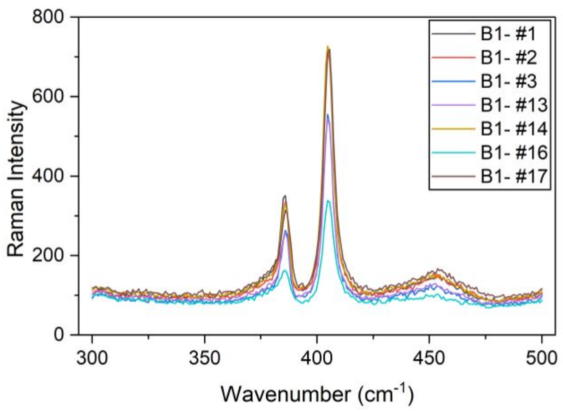

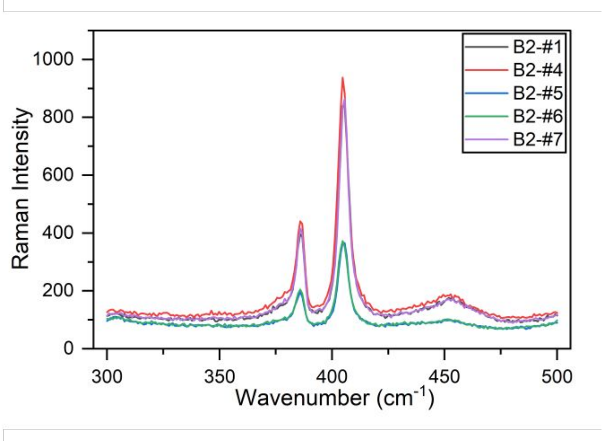

unit (cm^{-1})	B1-1	B1-2	B1-3	B1-13	B1-14	B1-16	B1-17	B2-1	B2-2	B2-4	B2-4	B2-5	B2-6	B2-7
E_{2g}	385.28	385.61	385.59	385.64	385.46	384.91	385.72	385.75	385.83	385.76	385.78	385.47	385.51	385.82
A_{1g}	404.9	405.09	404.94	404.95	404.89	405.12	405.24	405.28	405.28	404.88	404.85	405.07	404.84	405.25
Distance	19.62	19.48	19.35	19.31	19.43	20.21	19.52	19.53	19.45	19.12	19.07	19.6	19.33	19.43

Figure S 18 Raman spectra on one batch of Stack B (B1) and Control (B2) samples confirming the monolayer nature of the exfoliated films.

• Supplementary PL Spectra

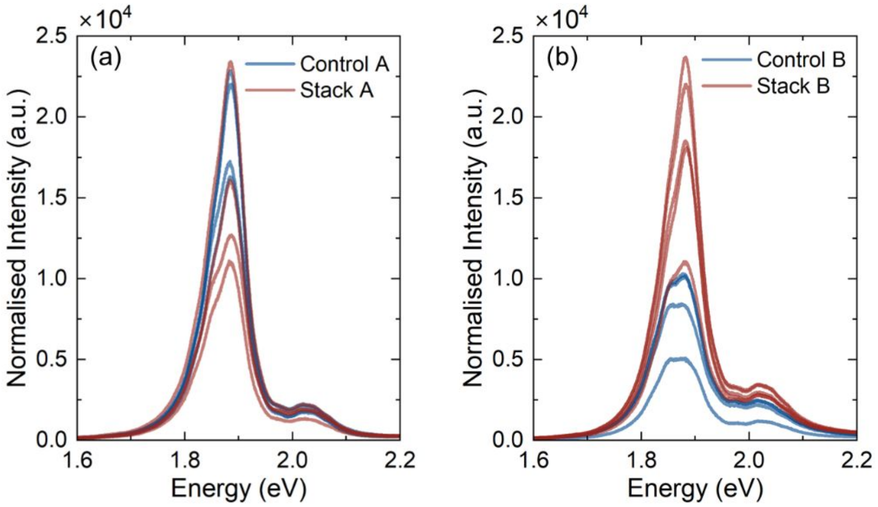

Figure S 19 Contact potential difference measured on a 3×3 cm^2 substrate charged for 5 minutes using the hot corona method at 450 °C. The substrate is later cut into 1×1 cm^2 samples to make Stack B devices. The same process was used to make all substrates for device fabrication.

• Details of Channel Characteristics Extraction

Transfer curves were analysed using MATLAB to extract the threshold voltage (V_{th}) and field-effect mobility (μ_{FE}). Forward and backward sweeps were treated separately. For fair comparison, all curves were fitted between V_{gs}=70-80 V despite their difference in the measurement range, using a linear extrapolation method. V_{th} was obtained from the intercept of the fitted line with the V_{gs} axis. Hysteresis (V_{th}) and μ_{FE} were analysed using data of the full measurement range using an automated sliding window method. Successive sections of the curve were fitted to a linear function and evaluated using the mean squared error. To ensure optimal fitting, the window size is set to 20-30 V for the forward curve, and 15-20 V for the backward curve. The window yielding the highest linearity was selected for parameter extraction. V_{hys} was determined from the difference between the backward and forward threshold voltage, while μ_{FE} is calculated from the slope of the forward transfer curve fit ($\partial I_{ds}/\partial V_{gs}$).

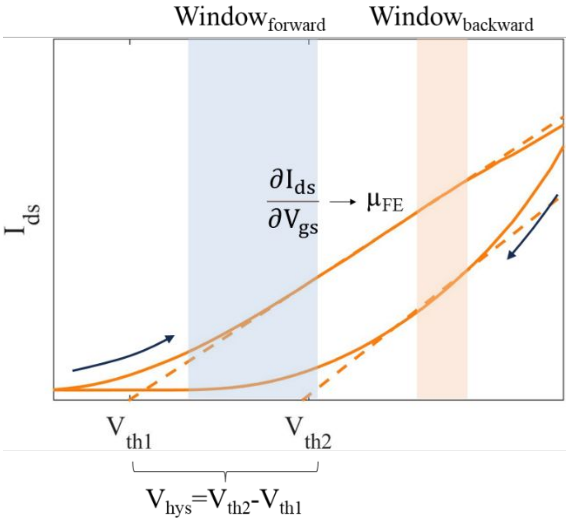

Figure S 20 Schematics of the fitting process of transfer curves to extract V_{hys} and μ_{FE}.

The extracted V_{hys} and μ_{FE} are shown in Figure S 21. It is noted that the same measurement range was applied to Control A and Stack A, yielding comparable V_{hys}, indicating a higher V_{hys} induced by the additional dielectric surface defect density. Meanwhile, a larger measurement range was applied on Stack B comparing to Control B, which contributes to the larger V_{hys}.

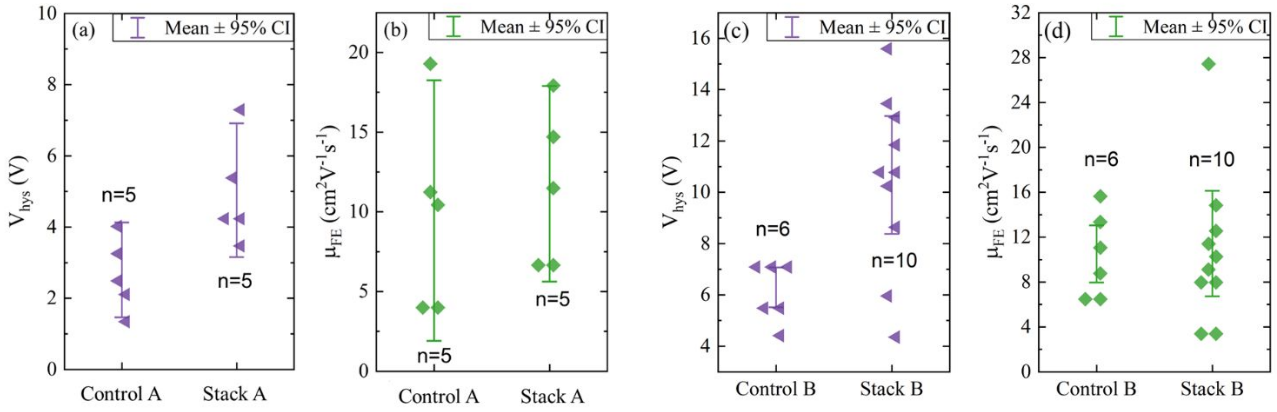

Figure S 21 The extracted (a,c) V_{hys} and (b,d) μ_{FE} from 2D MoS_2 FETs fabricated on (a,b) Control A, Stack A, (c,d) Control B and Stack B, respectively. The error bars represent the mean ± 95% confidence interval (CI).

• Additional PL Spectroscopy

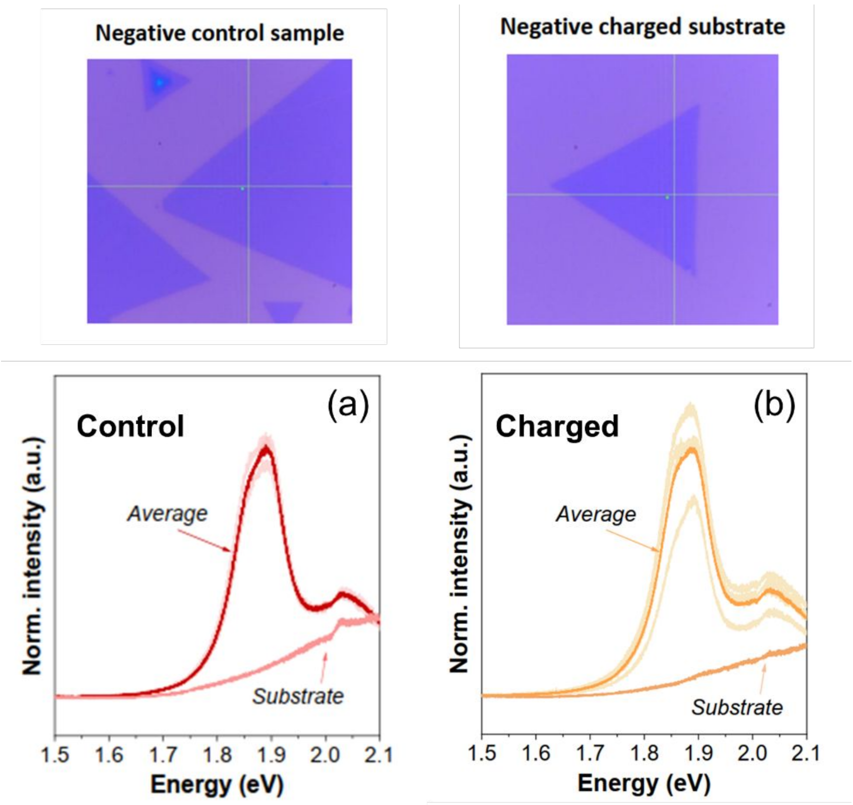

Figure S 22 Photoluminescence carried out on monolayer MoS_2 on (a) uncharged control capped with a HfO_x layer and (b) a substrate with hot corona charging after the deposition of a HfO_x capping layer and optical images of the transferred CVD-grown MoS_2 layers demonstrating the location for PL measurement.

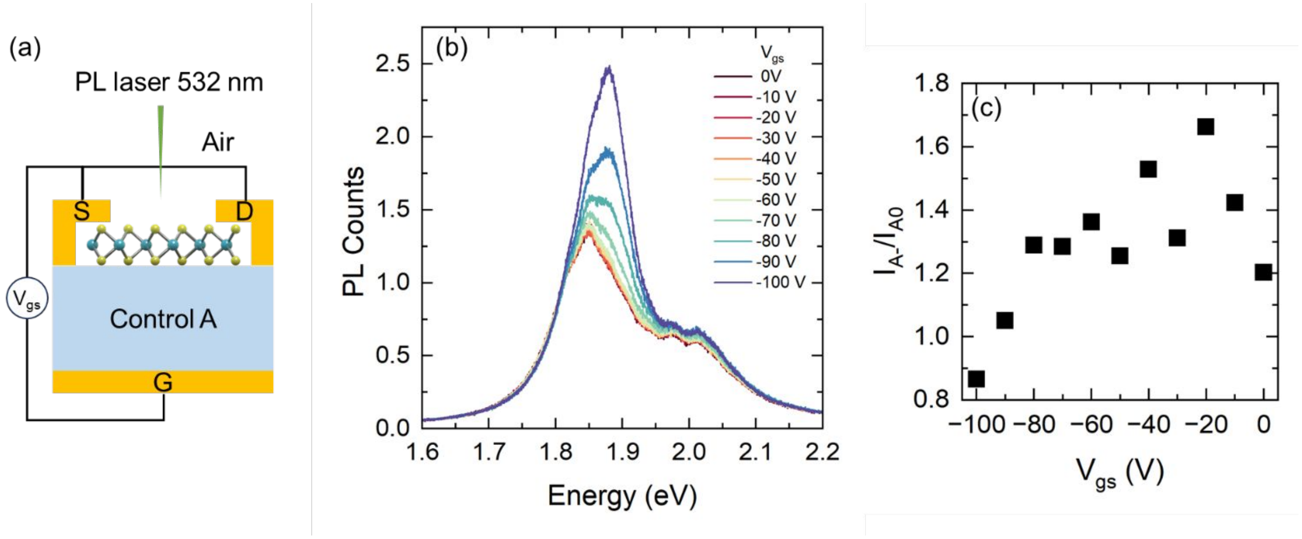

Figure S 23 (a) Experimental set-up of V_{gs}-dependent PL measurements. (b) Evolution of PL spectroscopy. (c) Extracted I_{A-}/I_{A0} values from PL spectra fitted using Lorentz functions.

Supporting Data for Electrostatic Coupling Simulations

The model parameters include the interface states density (D_{it}) and carrier capture cross sections ($\sigma_{n/p}$). A single value of $D_{it,mid}$ and is $\sigma_{n/p,mid}$ is defined for D_{it} and $\sigma_{n/p}$ at mid-gap, respectively. Near the band edges (ΔE<0.15 eV), tail distributions are introduced with peak values $D_{it,tail\text{-}don}$ ($D_{it,tail\text{-}acc}$) at the valence band (conduction band). These band-tail states are assigned capture cross sections $\sigma_{n/p,tail\text{-}don/acc}$. The

interface defects are acceptor-like in the upper half of the band gap (E-E_t>0 eV) and are donor-like in the lower half of the bandgap (E-E_t<0 eV). This model is based on previous work in.[7–9] For the calculation presented in this work, the capture cross sections were set to $\sigma_{n,mid}$=10^{-15} cm^2, $\sigma_{p,mid}$=10^{-16} cm^2, and $\sigma_{n/p,tail\text{-}don/acc}$=2×$10^{18}$ cm^2 and were kept constant across all simulations.

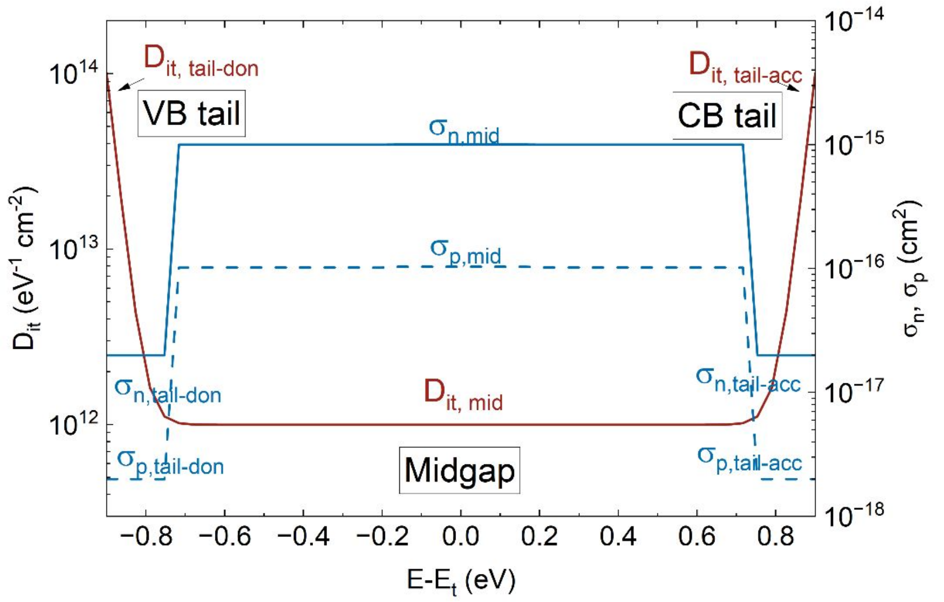

Figure S 24 Interface parameters used to calculate partition of embedded charge density Q_{surf} between interface charge density Q_{it} and channel charge density $Q_{channel}$ in this work.

Additional Information on Materials and Methods

• Details of Carrier Density Extraction from PL Spectra

The PL spectra were fitted using three Lorentz peaks. The integrated peak areas were used to estimate the electron density (n_{el}) in 2D MoS_2 based on mass action model describing the dynamic equilibrium between A^0, A^- and free electrons:[10]

$$n_{el} = \frac{I_{A^-}}{I_{A^0}} \times \frac{\gamma_{A^0}}{\gamma_{A^-}} \times \left[\left(\frac{4 m_{A^0} m_e}{\pi \hbar^2 m_{A^-}} \right) k_B\, T\, exp(-\frac{E_b}{k_B T}) \right]$$

where I_{A-} and I_{A0} are the area under peak A^- and A^0, γ_{A-} and γ_{A0} are the relative decay rates of A^- and A^0 excitons, and m_{A-} (1.15 m_0), m_{A0} (0.8 m_0) and m_{e-} (0.35 m_0) are the effective masses of A^-, A^0 and electrons respectively, where m_0 is the mass of free electrons. In this work, γ_{A-}/γ_{A0} is taken to be 0.15,[11] and the trion binding energy E_b is assumed to be 20 meV.[12]

References:

(1) Sze, S. M.; Ng, K. K. *Physics of Semiconductor Devices*; Wiley, 2006. https://doi.org/10.1002/0470068329.

(2) Gope, J.; Vandana; Batra, N.; Panigrahi, J.; Singh, R.; Maurya, K. K.; Srivastava, R.; Singh, P. K. Silicon Surface Passivation Using Thin HfO_2 Films by Atomic Layer Deposition. *Appl. Surf. Sci.* **2015**, *357*, 635–642. https://doi.org/10.1016/j.apsusc.2015.09.020.

(3) Lin, F.; Hoex, B.; Koh, Y. H.; Lin, J. J.; Aberle, A. G. Low-Temperature Surface Passivation of Moderately Doped Crystalline Silicon by Atomic-Layer-Deposited Hafnium Oxide Films. In *Energy Procedia*; 2012; Vol. 15, pp 84–90. https://doi.org/10.1016/j.egypro.2012.02.010.

(4) Dingemans, G.; van Helvoirt, C. A. A.; Pierreux, D.; Keuning, W.; Kessels, W. M. M. Plasma-Assisted ALD for the Conformal Deposition of SiO_2 : Process, Material and Electronic Properties. *J. Electrochem. Soc.* **2012**, *159* (3), H277–H285. https://doi.org/10.1149/2.067203jes.

(5) Bonilla, R. S.; Woodcock, F.; Wilshaw, P. R. Very Low Surface Recombination Velocity in N-Type c-Si Using Extrinsic Field Effect Passivation. *J. Appl. Phys.* **2014**, *116* (5). https://doi.org/10.1063/1.4892099.

(6) Thomas, N.; Mathew, S.; Nair, K. M.; O'Dowd, K.; Forouzandeh, P.; Goswami, A.; McGranaghan, G.; Pillai, S. C. 2D MoS_2: Structure, Mechanisms, and Photocatalytic Applications. *Materials Today Sustainability* **2021**, *13*, Art. no. 100073. https://doi.org/10.1016/j.mtsust.2021.100073.

(7) Bonilla, R. S.; Wilshaw, P. R. On the C-Si/SiO_2 Interface Recombination Parameters from Photo-Conductance Decay Measurements. *J. Appl. Phys.* **2017**, *121*, Art. no. 135301. https://doi.org/10.1063/1.4979722.

(8) Bonilla, R. S.; Al-Dhahir, I.; Yu, M.; Hamer, P.; Altermatt, P. P. Charge Fluctuations at the Si–SiO_2 Interface and Its Effect on Surface Recombination in Solar Cells. *Solar Energy Materials and Solar Cells* **2020**, *215*, Art. no. 110649. https://doi.org/10.1016/j.solmat.2020.110649.

(9) Girisch, R. B. M.; Mertens, R. P.; De Keersmaecker, R. F. Determination of Si-SiO_2 Interface Recombination Parameters Using a Gate-Controlled Point-Junction Diode Under Illumination. *IEEE Trans. Electron Devices* **1988**, *35* (2), 203–222. https://doi.org/10.1109/16.2441.

(10) Liu, Y.; Shen, T.; Linghu, S.; Zhu, R.; Gu, F. Electrostatic Control of Photoluminescence from A and B Excitons in Monolayer Molybdenum Disulfide. *Nanoscale Adv.* **2022**, *4*, 2484–2493. https://doi.org/10.1039/d2na00071g.

(11) Mouri, S.; Miyauchi, Y.; Matsuda, K. Tunable Photoluminescence of Monolayer MoS_2 via Chemical Doping. *Nano Lett.* **2013**, *13*, 5944–5948. https://doi.org/10.1021/nl403036h.

(12) Mak, K. F.; He, K.; Lee, C.; Lee, G. H.; Hone, J.; Heinz, T. F.; Shan, J. Tightly Bound Trions in Monolayer MoS_2. *Nat. Mater.* **2013**, *12*, 207–211. https://doi.org/10.1038/nmat3505.
